# Gut Microbiota Dynamics during Chemotherapy in Epithelial Ovarian Cancer Patients Are Related to Therapeutic Outcome

**DOI:** 10.3390/cancers13163999

**Published:** 2021-08-08

**Authors:** Federica D’Amico, Anna Myriam Perrone, Simone Rampelli, Sara Coluccelli, Monica Barone, Gloria Ravegnini, Marco Fabbrini, Patrizia Brigidi, Pierandrea De Iaco, Silvia Turroni

**Affiliations:** 1 Department of Medical and Surgical Sciences, University of Bologna, 40138 Bologna, Italy; myriam.perrone@aosp.bo.it (A.M.P.); sara.coluccelli2@unibo.it (S.C.); monica.barone@unibo.it (M.B.); patrizia.brigidi@unibo.it (P.B.); pierandrea.deiaco@unibo.it (P.D.I.); 2 Department of Pharmacy and Biotechnology, University of Bologna, 40126 Bologna, Italy; simone.rampelli@unibo.it (S.R.); gloria.ravegnini2@unibo.it (G.R.); m.fabbrini@unibo.it (M.F.); silvia.turroni@unibo.it (S.T.); 3 Division of Oncologic Gynecology, IRCCS Azienda Ospedaliero, University of Bologna, 40138 Bologna, Italy; 4 Centro di Studio e Ricerca delle Neoplasie Ginecologiche (CSR), University of Bologna, 40138 Bologna, Italy

**Keywords:** gut microbiota, epithelial ovarian cancer, chemotherapy, neoadjuvant therapy, adjuvant therapy, platinum resistance, platinum sensitivity, lactate, 16S rRNA gene sequencing, inferred metagenomics

## Abstract

**Simple Summary:**

This pilot study on the trajectory of the gut microbiota (GM) in patients with epithelial ovarian cancer undergoing neoadjuvant and adjuvant chemotherapy highlighted peculiar dynamics associated with the therapeutic outcome. In particular, platinum-resistant patients showed a marked temporal reduction in GM diversity and increased instability with loss of health-associated taxa and increased proportions of lactate-producing microorganisms compared to those sensitive to platinum. These potential GM signatures of therapeutic failure are detectable within the first half of chemotherapy cycles, suggesting that early integrated treatments also aimed at modulating GM could influence therapeutic outcome. Further studies in larger cohorts combining multiple omics and possibly animal models are urgently needed for in-depth mechanistic understanding.

**Abstract:**

Epithelial ovarian cancer (EOC) is one of the most lethal and silent gynecological tumors. Despite appropriate surgery and chemotherapy, relapse occurs in over half of patients with a poor prognosis. Recently, the gut microbiota (GM) was hypothesized to influence the efficacy of anticancer therapies, but no data are available in EOC. Here, by 16S rRNA gene sequencing and inferred metagenomics, we profiled the GM of EOC patients at diagnosis and reconstructed its trajectory along the course of neoadjuvant or adjuvant chemotherapy up to follow-up. Compared to healthy subjects, the GM of EOC patients appeared unbalanced and severely affected by chemotherapy. Strikingly, discriminating patterns were identified in relation to the therapeutic response. Platinum-resistant patients showed a marked temporal reduction in GM diversity and increased instability with loss of health-associated taxa and increased proportions of *Coriobacteriaceae* and *Bifidobacterium*. Notably, most of these microorganisms are lactate producers, suggesting increased lactate production as supported by inferred metagenomics. In contrast, the GM of platinum-sensitive patients appeared overall more diverse and stable and enriched in lactate utilizers from the *Veillonellaceae* family. In conclusion, we identified potential GM signatures of therapeutic outcome in EOC patients, which could open up new opportunities for cancer prognosis and treatment.

## 1. Introduction

Epithelial ovarian cancer (EOC) is a relatively rare disease whose incidence rate is very high in western countries, such as Europe and North America, with eight cases out of 100,000 [1,2]. Currently, there is no established screening test for this disease, and this cancer represents the most lethal and silent gynecological tumor with diagnosis in advanced stages (III–IV) in about 65% of cases and a 5-year relative survival of only 20–30% [3]. Primary tumors originate from the epithelium of the ovary, the fallopian tube, or the peritoneum and then spread to the peritoneal surfaces and viscera of the pelvis and the whole abdomen (carcinosis). The cancer’s spread by blood and lymphatic way is a rare event [4]. The standard therapeutic approach is surgical cytoreduction followed by first-line standard chemotherapy with platinum and taxane compounds. When surgery is not possible for disease extent, neoadjuvant chemotherapy is an option to reduce the burden of the disease and achieve cytoreduction in responders; in non-responders, the prognosis is poor. Despite optimal surgery and appropriate first-line chemotherapy, about 70–80% of patients with EOC develop disease recurrence, and patients are candidates to new therapeutic opportunities, such as novel drug classes combined with different schemes of chemotherapy [5]. When EOC recurs, the prognosis is very poor. Recurrence occurs in about 23% of patients during or within 6 months after first-line chemotherapy (platinum-resistant, PR) and 60% after 6 months (platinum-sensitive, PS). Progressively, PS patients experience shorter disease-free intervals, eventually becoming PR [6]. Pathogenesis of EOC is poorly understood. Risk factors are represented by age, late menopause, genetic factors (breast cancer gene (BRCA) mutation and Lynch syndrome), and environmental agents. The role of infections and inflammation in the pathogenesis of EOC is not completely elucidated; it is likely that inflammation and ovulation with changes in hormone levels lead to DNA damage by oxidative stress. Pelvic inflammatory disease, although not a recognized risk factor, is associated with EOC in several publications. The correlation between some agents such as *Chlamydia*, HPV, and cytomegalovirus infections was investigated, but it is difficult to make any firm conclusion. Interestingly, studies by Banerjee et al. [7] profiled the EOC oncobiome, showing distinct viral, bacterial, and fungal signatures compared to matched and non-matched controls, including increased representation or unique detection of retroviridiae and HPV, several members of Proteobacteria and Firmicutes, yeasts, and zygomycetous fungi. Although it is not yet clear whether these microorganisms are actually involved in tumor pathogenesis, the manipulation of human microbiomes was recently advanced as a strategy to affect tumor progression and response to therapies [8,9,10,11]. In this scenario, special attention is paid to the gut microbiota (GM), i.e., the richest and the most diverse microbial community of the human holobiont closely linked to our health [12]. Due to its ability to modulate immune responses and interfere with drug metabolism [13], GM is indeed a very hot topic in cancer research [14,15,16]. In particular, since the first landmark studies in animal models [17,18], an ever-growing body of evidence indicates that peculiar GM profiles can improve the efficacy of anticancer therapy while reducing side effects. These “more favorable” profiles appear to share greater diversity and greater proportions of health-associated microorganisms, mainly producers of short-chain fatty acids (SCFAs) [10,19]. However, the vast majority of studies were conducted in patients with hematological malignancies or melanoma, while, to date, no information is available on the possible role of GM in EOC.

In an attempt to bridge this gap, here, we prospectively profiled the GM of 24 women with EOC through 16S rRNA gene sequencing and reconstructed its trajectories over the course of chemotherapy in relation to the therapeutic response (PR vs. PS). We recruited patients with high-grade papillary serous carcinoma, which is the most frequent histotype involved in carcinosis. Fecal samples were collected at diagnosis and before and after each chemotherapeutic cycle until follow-up over a period of about 1.5 years. The data herein generated strongly suggest that the GM dynamics during chemotherapy could serve as a prognostic biomarker and innovative therapeutic target for EOC.

## 2. Materials and Methods

### 2.1. Patient Enrollment and Fecal Sampling

Patients with EOC diagnosis referred to the Division of Oncologic Gynecology, IRCCS Azienda Ospedaliero-Universitaria di Bologna (Bologna, Italy) were enrolled for a pilot longitudinal study on GM from diagnosis (T0) through chemotherapy treatment (pre and post each cycle performed every 3 weeks) until the follow-up. The local Ethical Committee of Area Vasta Emilia Romagna approved the study (CE Emilia Area Vasta N. 122/2017/O/Tess). Written informed consent was obtained from each enrolled patient during the first access to the Gynecologic Oncology Unit. Inclusion criteria were: (i) suspicious or confirmed first diagnosis of ovarian cancer; (ii) patients treated at Gynecologic Oncology Unit; and (iii) patients available to collect stool samples at diagnosis, during treatment, and at follow-up. Patients with carcinosis or metastasis from other organs, inflammatory bowel disease, and chronic antibiotic intake were excluded from the study. All enrolled patients were clustered in two groups (12 vs. 12 subjects) depending on the therapeutic program: (i) women with moderate disease extension were treated by surgical cytoreduction followed by first-line standard chemotherapy with platinum and taxane compounds (adjuvant group); (ii) patients with extensive disease were treated with neoadjuvant chemotherapy with platinum and taxane compounds before surgical cytoreduction (neoadjuvant group). Evaluable data were age, BMI, personal clinical history, histological types, International Federation of Gynecology and Obstetrics (FIGO) stage, number and doses of chemotherapy cycles, and surgical information including extension of disease (peritoneal cancer index, PCI) (Table 1). During the study, patients were asked to report any other concomitant therapies. At the end of both treatments (i.e., adjuvant and neoadjuvant chemotherapy), follow-up was performed as follows in order to detect relapse: CA 125 examination and clinical assessment every 4 months for the first 2 years and then every 6 months for 5 years with a CT scan every 6 months. During the post-treatment revaluations, the EOC subjects for both groups were further divided into two sub-groups based on the time elapsed between the last platinum intake and the diagnosis of relapse: (i) fewer than 6 months (platinum-resistant; PR), or (ii) more than 6 months (platinum-sensitive; PS). Fecal samples were collected at diagnosis from all patients (*n* = 24) and before and after each chemotherapy cycle (*n* = 344). Specifically, the last/first stools were collected in the 3 days before/after each cycle. For follow-up, samples were taken every 3 months in all patients with no signs of relapse (*n* = 38). A total of 406 fecal samples were thus collected, stored at −80 °C, and shipped on dry ice to the Department of Pharmacy and Biotechnology, University of Bologna (Bologna, Italy) for GM analysis.

### 2.2. Microbial DNA Extraction

Two hundred and fifty milligrams of fecal sample were used for microbial DNA extraction through a method combining bead-beating and column purification, as described in Yu and Morrison [20]. The following modifications were introduced: 1 mL of lysis buffer (500 mM NaCl, 50 mM Tris-HCl, pH 8, 50 mM EDTA, and 4% SDS), four 3 mm glass beads, and 0.5 g of 0.1 mm zirconia beads (BioSpec Products, Bartlesville, OK, USA) were used to perform chemical and mechanical lysis of the samples in a FastPrep instrument (MP Biomedicals, Irvine, CA, USA) at 5.5 movements/s for 1 min, repeated three times [21]. Samples were incubated at 95 °C for 15 min and then centrifuged at 13,000 rpm for 5 min. Subsequently, the supernatant was added with 10 M ammonium acetate and centrifuged for 10 min at 13,000 rpm. The supernatants were then incubated in ice for 30 min with one volume of isopropanol for nucleic acid precipitation. A washing step with 70% ethanol was performed, and the precipitated nucleic acids were resuspended in 100 μL of TE buffer (10 mM Tris-HCl, 1 mM EDTA pH 8.0). Two microliters of 10 mg/mL DNase-free RNase were then added, and the samples were incubated at 37 °C for 15 min. Finally, a column-based method was used for DNA purification using the DNeasy Blood and Tissue Kit (QIAGEN, Hilden, Germany) as per manufacturer’s instructions. The yield and the quality of the extracted DNA were assessed with a NanoDrop ND-1000 spectrophotometer (NanoDrop Technologies, Wilmington, DE, USA).

### 2.3. 16S rRNA Gene Amplification and Sequencing

The V3–V4 hypervariable regions of the 16S rRNA gene were amplified with primers 341F and 785R, including Illumina adapter overhang sequences, as described in Rampelli et al. [21]. For library preparation, the following steps were performed: magnetic bead-based amplicon clean-up (Agencourt AMPure XP, Beckman Coulter, Brea, CA, USA), limited-cycle PCR using Nextera technology to index libraries, and another clean-up as above. Indexed libraries were pooled at an equimolar concentration of 4 nM, denatured, and diluted to 5 pM prior to sequencing on an Illumina MiSeq platform with a 2 × 250 bp paired-end protocol per manufacturer’s instructions (Illumina, San Diego, CA, USA).

### 2.4. Bioinformatics and Statistics

All sequences were processed using a pipeline that combined PANDASeq [22] and QIIME 2 [23]. After filtering the reads by length and quality, the DADA2 pipeline was used to bin the remaining reads into amplicon sequence variants (ASVs) [24]. Taxonomic classification was performed using the VSEARCH algorithm [25] on the Greengenes database (May 2013 release). Chimeras were removed during the bioinformatic steps. Metagenome prediction of Greengenes-picked ASVs was performed with PICRUSt2 [26] using MetaCyc [27] as a reference for pathway annotation. Publicly available sequences of healthy women matched to EOC patients for several GM-associated confounding factors (i.e., age, BMI, and geography) were downloaded and used as a control. Sequences were from different cohorts to minimize study-related bias, specifically from: De Filippis et al. [28] (deposited in NCBI SRA: Bioproject ID SRP042234), Schnorr et al. [29] (Italian samples; MG-RAST database: project ID mgp12183), and Biagi et al. [30] (elderly samples; MG-RAST database: project ID mgp17761). Alpha diversity was assessed using the number of observed ASVs and the Simpson inverse index. Bray–Curtis and weighted and unweighted UniFrac distances were used to build principal coordinates analysis (PCoA) graphs. All statistical analyses were carried out with the R software. PCoA plots were generated using the “vegan” (Available online: http://www.cran.r-project.org/package=vegan/, accessed on 29 April 2020) and “Made4” [31] packages, and data separation was tested by a permutation test with pseudo-F ratio (function “Adonis” in “vegan”). To assess differences in diversity and GM composition at different taxonomic levels among groups, Kruskal–Wallis test followed by post-hoc Wilcoxon test were used. Discriminating taxa and MetaCyc pathways between groups were identified through linear discriminant analysis (LDA) effect size (LEfSe) algorithm [32]. Only taxa with LDA score > 2 at *p* < 0.05 were considered discriminating. For the generation of co-abundance groups (CAGs), only bacterial genera present in at least 10% of the samples were considered. As previously shown in Claesson et al. [33], associations among genera were assessed using the Kendall correlation test, visualized using hierarchical Ward-linkage clustering based on Spearman correlation coefficients, and used to define CAGs. Wiggum plot networks were created using Cytoscape software [34] with the circle size proportional to the relative bacterial abundance and the connection between nodes representing significant Kendall correlations between genera (*p* < 0.05). Progression-free survival and overall survival according to GM were estimated using the Kaplan–Meier method, and comparisons were made using the log-rank test. As GM variables, alpha diversity (as number of observed ASVs) and relative abundance of discriminating taxa were considered, and the cohort was stratified using the median of each variable as previously shown [35,36]. As for the time-point, based on the differences that emerged between PR and PS patients, samples were selected halfway through the chemotherapy treatment (i.e., before cycle 7 for neoadjuvant therapy and before cycle 4 for adjuvant therapy) (see also Results). Power calculation was computed with micropower R package [37]; we found that the size of PS and PR groups allowed 90% power to detect an ω2 of 0.003. When necessary, p values were corrected for multiple comparisons using the Benjamini–Hochberg method. A false discovery rate (FDR) ≤ 0.05 was considered as statistically significant. FDR ≤ 0.1 was considered as a trend.

## 3. Results

### 3.1. Study Cohort Description

Twenty-four women affected by high-grade serous EOC were enrolled at the Division of Oncologic Gynecology, IRCCS Azienda Ospedaliero-Universitaria di Bologna, Bologna, Italy. Patient characteristics are shown in Table 1. The median age was 57 years (range, 39–71) and body mass index (BMI) 22 kg/m^2^ (range, 19–34). Twenty-two (92%) patients experienced advanced FIGO stage (III–IV), and nine (37%) patients reported germinal BRCA mutation. All patients received a median of six cycles of carboplatin and taxane (range, 6–15), carcinomatosis was present in all patients except one case of stage IC, and peritoneal cancer index (PCI) showed a median of 20 (range, 0–37). Twelve patients received neoadjuvant chemotherapy because they were judged not optimally cytoreducible at diagnostic laparoscopy [5], and 12 received up-front surgery. No differences were found in age, BMI, FIGO stage, or BRCA mutation between the neoadjuvant and the adjuvant groups, although neoadjuvant-treated patients were characterized by higher PCI (median in neoadjuvant vs. adjuvant group, 23 vs. 18) (Wilcoxon test, *p* = 0.0044), number of cycles received (9 vs. 6) (*p* = 0.0477), and levels of CA 125 (1040 vs. 845) (*p* = 0.0486). In the neoadjuvant group, six patients were judged platinum-resistant (PR) and six platinum-sensitive (PS) according to relapse and residual disease after surgery. On the other hand, in the adjuvant group, three patients were grouped as PR and nine as PS. When we compared PR and PS patients, no differences in age, BMI, or number of chemotherapy cycles were observed, while PCI (median in PR vs. PS patients, 24.5 vs. 22.5) (*p* = 0.0579) and CA 125 (1115 vs. 1024.5) (*p* = 0.0023) levels tended to be or were higher in PR patients of the neoadjuvant group. Median progression-free survival (PFS) and overall survival (OS) in the whole cohort were 14 (range, 1–29) and 16 months (8–39), respectively.

**Figure 1:**
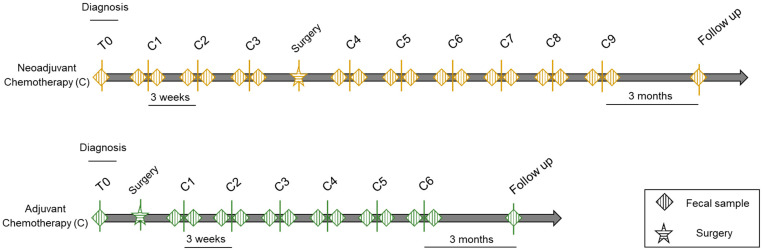
Study design. Schematic representation of fecal sampling for patients with epithelial ovarian cancer given neoadjuvant (top) or adjuvant (bottom) chemotherapy. Diamonds indicate sampling time-points, i.e., at diagnosis (T0), before and after each chemotherapy cycle (C), and at follow-ups every 3 months from the end of therapy. Chemotherapy courses were repeated every 3 weeks depending on the tumor response.

For each patient, fecal sampling took place at several time-points starting from diagnosis (T0), before and after each chemotherapy cycle (C, pre and post), and at follow-ups every 3 months from the end of therapy for a total of 406 fecal samples over a study period of approximately 1.5 years (Figure 1). Fecal samples were subjected to 16S rRNA gene sequencing, yielding 12,399,150 high-quality reads (mean ± SD, 30,540 ± 21,518).

### 3.2. The Gut Microbiota in EOC Patients at Diagnosis

**Figure 2:**
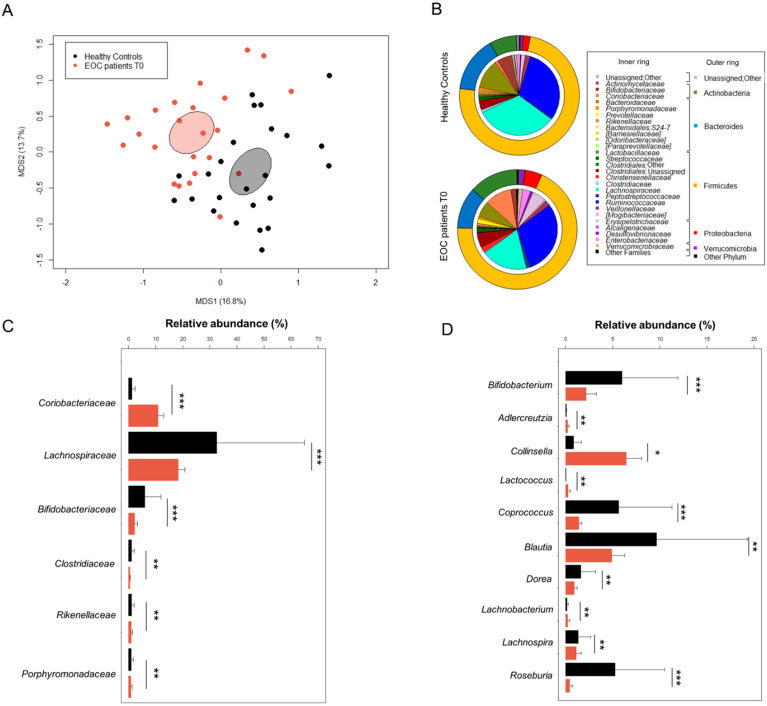
The gut microbiota of EOC patients at diagnosis compared to healthy women. (**A**) PCoA based on Bray–Curtis dissimilarity between the genus-level profiles of 24 EOC patients and 24 healthy age/BMI-matched women living in the same geographical area (across Italy). A significant separation between groups was found (permutation test with pseudo-F ratio, *p* = 0.001). Ellipses include 95% confidence area based on the standard error of the weighted average of sample coordinates. (**B**) Pie charts showing the average relative abundance of major phyla (outer ring) and families (inner ring) in the gut microbiota of EOC patients and healthy women. Only taxa with relative abundance > 0.1% in at least one sample are shown. Relative abundance (mean ± SEM) of families (**C**) and genera (**D**) differentially represented between groups (Wilcoxon test, * for *p* ≤ 0.05; ** for *p* < 0.01; *** for *p* < 0.001). Only families with mean relative abundance > 0.5% in at least one of the two groups are shown.

The GM of 24 EOC patients at diagnosis (T0) was profiled and compared with that of 24 healthy women from previous studies [28,29,30] matched by several microbiota-associated confounding variables (i.e., age, BMI, and geography) [38]. No difference in alpha diversity was observed between two groups using the Simpson’s inverse index (Wilcoxon test, *p* = 0.3). In contrast, Bray–Curtis distance-based principal coordinates analysis (PCoA) showed significant segregation between the GM of EOC patients and that of healthy subjects (permutation test with pseudo-F ratio, *p* = 0.001) (Figure 2A). Regarding the taxonomic composition, the GM of both patients and controls was dominated by the phylum Firmicutes (mean relative abundance in patients vs. controls, 68.8% vs. 73.9%) along with Bacteroidetes (11.2% vs. 15.1%), Actinobacteria (13.2% vs. 7.1%), and Proteobacteria (4.6% vs. 1.9%) (Figure 2B). Consistent with the typical adult-like GM profile [39], *Ruminococcaceae* (29.4% vs. 32.5%), *Lachnospiraceae* (18.3% vs. 32.5%), and *Bacteroidaceae* (6.3% vs. 11.7%) were the most abundant families. However, EOC patients were characterized by decreased proportions of *Lachnospiraceae* (Wilcoxon test, *p* < 0.001) as well as other families such as *Bifidobacteriaceae*, *Clostridiaceae*, *Rikenellaceae*, and *Porphyromonadaceae* (*p* ≤ 0.01). On the other hand, the EOC-related GM profiles were markedly enriched in *Coriobacteriaceae* (*p* < 0.001) (Figure 2C). Additionally, at the genus level, several differences emerged between the two groups (Figure 2D). In particular, compared to healthy subjects, the GM of EOC patients was enriched in *Coriobacteriaceae* members, *Adlercreutzia* and *Collinsella* (*p* ≤ 0.05), as well as in *Lactococcus* and *Lachnobacterium* (*p* ≤ 0.01), while it was depleted in several *Lachnospiraceae* genera such as *Coprococcus*, *Blautia*, *Dorea*, *Lachnospira*, and *Roseburia* along with *Bifidobacterium* (*p* ≤ 0.01).

### 3.3. Gut Microbiota Dynamics in EOC Patients during Chemotherapy

The dynamics of GM in 24 women with EOC were reconstructed during adjuvant and neoadjuvant treatments (12 vs. 12 patients) from baseline (T0) through each chemotherapy cycle (C, pre and post) until follow-up. For both patient groups, alpha diversity was found to fluctuate over time with, in particular, a significant reduction from the baseline during the first few cycles of chemotherapy treatments (preC3 for the adjuvant group and preC4 for the neoadjuvant group) (Wilcoxon test, *p* ≤ 0.1) (Figure S1). PCoA based on weighted UniFrac distances showed no separation between groups nor within each group over time (permutation test with pseudo-F ratio, *p* > 0.05) but strong individuality, with samples from the same subject clustering nearby for both treatment groups (*p* = 0.001) (Figure S1). As previously shown at baseline, the GM was dominated by the phylum Firmicutes at all time-points considered regardless of the treatment group with varying proportions of Bacteroidetes, Actinobacteria, Proteobacteria, and Verrucomicrobia. Compared to T0, patients receiving neoadjuvant therapy showed a significant increase in Actinobacteria over time (especially in samples preC4, postC6, and postC7), while those given adjuvant therapy showed a significant reduction in Firmicutes in the first few cycles (preC3 and postC4) (Wilcoxon test, *p* < 0.05). Consistent results were obtained at the family level with an increase over time in the relative abundance of *Coriobacteriaceae* in patients undergoing neoadjuvant chemotherapy accompanied by a reduction in *Ruminococcaceae*, especially *Faecalibacterium* and *Ruminococcus*, almost after every chemotherapy cycle (*p* < 0.05). For patients given adjuvant therapy, we mainly observed a reduction in *Lachnospiraceae* (preC5 and preC7, *p* < 0.05) (Figure S2).

### 3.4. Potential Gut Microbiota Signatures of Therapeutic Response

**Figure 3:**
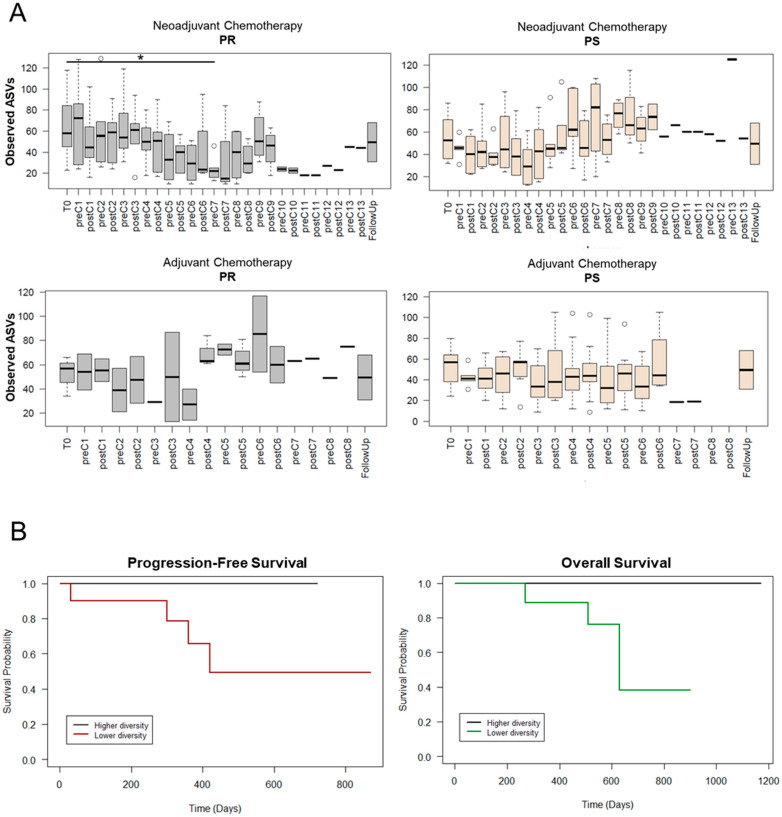
Dynamics of alpha diversity in EOC patients during chemotherapy based on therapeutic response and association with survival. (**A**) Boxplots showing the distribution of the number of observed ASVs during neoadjuvant (top) or adjuvant (bottom) chemotherapy. Patients were stratified by response to therapy: PR, platinum-resistant (left) vs. PS, platinum-sensitive (right). C, chemotherapy cycle. Wilcoxon test, * for *p* ≤ 0.05. (**B**) Kaplan–Meier curves for progression-free survival (left) and overall survival (right) in the whole cohort. EOC patients were stratified by higher or lower number of observed ASVs (relative to median) at the preC7 (for neoadjuvant therapy) or the preC4 (for adjuvant therapy) time-points. Log-rank test, *p* ≤ 0.1.

Within each treatment group, patients were stratified by platinum sensitivity (PS) or resistance (PR) based on the time before relapses appeared (beyond or within 6 months of first-line chemotherapy, respectively), and their GM profiles were followed over time. As discussed above for the whole cohort, an overall trend towards reduced alpha diversity in the first treatment cycles was observed across all study groups (Figure 3A). This was particularly evident for PR patients receiving neoadjuvant therapy for whom GM diversity dropped dramatically before the C7 cycle (Wilcoxon test, *p* = 0.03). Interestingly, these low levels tended to persist over time unlike what was observed for PS patients given neoadjuvant therapy, whose values remained substantially high until follow-up. A similar pattern (i.e., almost no change in the PS group with greater fluctuations in the PR group) was observed in EOC patients receiving adjuvant treatment. In particular, although not significant, the lowest alpha diversity value for PR patients receiving adjuvant therapy was observed at about half of the treatment (i.e., preC4). Based on these considerations, the number of observed ASVs in preC7 samples for neoadjuvant therapy and preC4 samples for adjuvant treatment was used to evaluate associations with progression-free survival and overall survival in the whole cohort. As shown in the Kaplan–Meier curves of Figure 3B, higher diversity at these time-points tended to be associated with longer survival (both progression-free and overall) (log-rank test, *p* ≤ 0.1).

**Figure 4:**
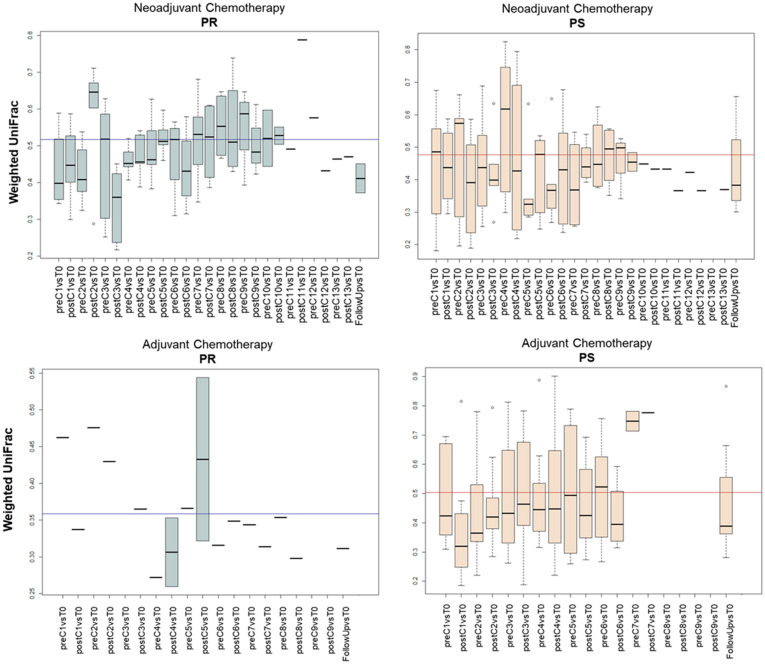
Dynamics of beta diversity in EOC patients during chemotherapy based on therapeutic response. Boxplots show the distribution of weighted UniFrac distances between each time-point and the respective T0 for each EOC patient during neoadjuvant (top) or adjuvant (bottom) chemotherapy. Patients were stratified by response to therapy: PR, platinum-resistant (left) vs. PS, platinum-sensitive (right). The mean inter-patient variability at baseline (i.e., T0) is depicted as a line in each plot. C, chemotherapy cycle.

As for beta diversity, no intra-group separation over time was evident in the weighted UniFrac-based PCoA plot (permutation test with pseudo-F ratio, *p* > 0.05), but there was in the samples segregated by therapeutic response, i.e., there was significant separation between all samples from PR patients and those from PS patients for both treatment groups (neoadjuvant group: *p* = 0.009; adjuvant group: *p* = 0.001) (Figure S3). Noteworthy is that such a separation was not significant at diagnosis (*p* > 0.05) (Figure S4). In order to further explore the GM structural variation, we calculated the weighted UniFrac distances between each time-point and the respective T0 for all PS and PR patients during both chemotherapy treatments (i.e., neoadjuvant vs. adjuvant) (Figure 4). Interestingly, the GM underwent more or less extensive fluctuations depending on the study group. In particular, for PR patients undergoing neoadjuvant treatment, the distance from T0 increased before the C7 cycle, then remained above the baseline inter-individual variation to drop only in the last time-points and in the follow-up. In contrast, the variations in the corresponding PS group were mostly lower than the inter-patient variability at T0. Similarly, for adjuvant treatment, the distance values for PR patients were often greater than the average inter-patient variation at baseline, while the GM variation in PS patients remained nearly constant over time. Taken together, these data suggest greater GM resistance to chemotherapy-related perturbations for PS patients with greater temporal instability with loss of individual fingerprint for PR patients.

**Figure 5:**
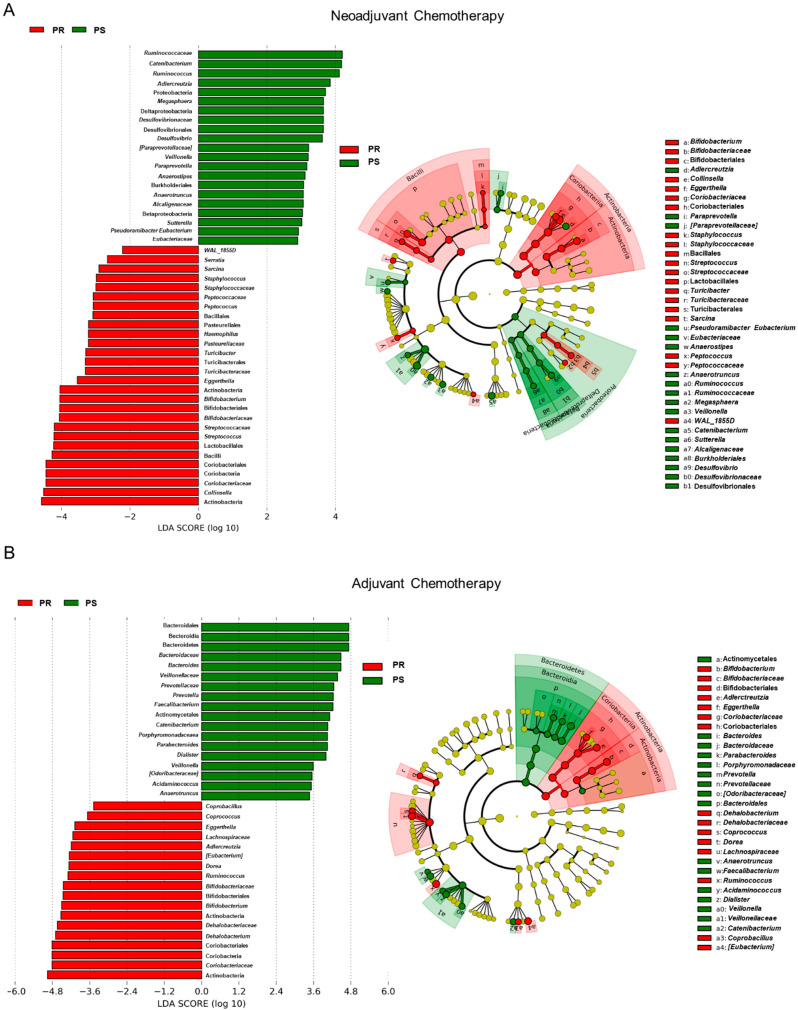
Potential taxonomic signatures of therapeutic response in EOC patients receiving neoadjuvant or adjuvant chemotherapy. Differentially represented taxa between platinum-sensitive (i.e., PS) and platinum-resistant (i.e., PR) EOC patients receiving neoadjuvant (**A**) or adjuvant (**B**) chemotherapy were identified by linear discriminant analysis (LDA) effect size (LEfSe) analysis. Left, LDA scores; right, cladograms. The logarithmic threshold for discriminative features was set to 2.0. See also Figure S5.

In an attempt to identify potential taxonomic markers of platinum response, a linear discriminant analysis (LDA) effect size (LEfSe) analysis was performed (Figure 5). Members of Actinobacteria, including the *Coriobacteriaceae* family with *Eggerthella* and unclassified genera and *Bifidobacterium*, were found to discriminate for PR patients regardless of chemotherapy group (i.e., neoadjuvant vs. adjuvant). On the other hand, the GM of PS patients was discriminated by *Veillonellaceae* members (i.e., *Veillonella*, *Megasphaera*, and *Dialister*) as well as by *Catenibacterium* and *Anaerotruncus*. As for peculiarities of each treatment group, Bacteroidetes members (*Bacteroides*, *Prevotella*, and *Parabacteroides*), *Faecalibacterium*, and *Acidaminococcus* were characteristic of PS patients undergoing adjuvant chemotherapy, while *Desulfovibrio*, *Paraprevotella*, *Anaerostipes*, *Sutterella*, and *Pseudoramibacter_Eubacterium* were characteristic of those receiving the neoadjuvant treatment. Furthermore, the GM of PR patients of the neoadjuvant group was specifically discriminated by *Serratia*, *Sarcina*, *Staphylococcus*, *Peptococcus*, *Haemophilus*, *Turicibacter*, *Streptococcus*, and *Collinsella*, while that of PR patients receiving adjuvant therapy was discriminated by *Coprobacillus*, *Coprococcus*, *[Eubacterium]*, *Dorea*, and *Dehalobacterium*. The temporal dynamics of the main taxa identified as discriminating between PR and PS patients are reported in Figure S5. Among these, it is worth noting that the proportions of *Coriobacteriaceae*, especially *Eggerthella*, and *Bifidobacterium* were overall considerably higher in PR than in PS patients throughout the course of treatment. In contrast, *Veillonellaceae* members, *Catenibacterium*, and *Anaerotruncus* were poorly represented if not absent in the GM of PR patients at the different time-points while reaching high relative abundance values in PS patients during therapy. Consistently, Kaplan–Meier survival curves showed that higher relative abundance of *Coriobacteriaceae* halfway through the chemotherapy treatment was associated, albeit not significantly, with lower survival probability, while an opposite trend was observed for *Veillonellaceae* (log-rank test, *p* ≤ 0.1) (Figure S6). It should be noted that all the taxa mentioned above were not differentially represented at baseline between PR and PS patients receiving neoadjuvant or adjuvant therapy (Wilcoxon test, *p* > 0.05) and that some differences, particularly the overabundance of *Eggerthella* in PR patients (follow-up vs. baseline, neoadjuvant group: *p* = 0.03) and *Dialister* in PS patients (adjuvant group: *p* = 0.1), persisted or tended to persist in the first follow-up 3 months after the end of the therapy. Six months after the end of the treatments, the proportions of *Dialister* in PS patients who received adjuvant chemotherapy tended to remain higher (*p* = 0.1), while in PS patients of the neoadjuvant group, there were significant or near-significant increases in some PR-associated microbial signatures, i.e., *Eggerthella* (*p* = 0.1) and *Adlercreutzia* (*p* = 0.04) (Figure S7). Supporting an association between these potential GM signatures of platinum response and therapeutic outcome, 67% of PS patients who received neoadjuvant chemotherapy relapsed at 6 months of follow-up compared to 44% of PS patients of the adjuvant group (Table 1).

**Figure 6:**
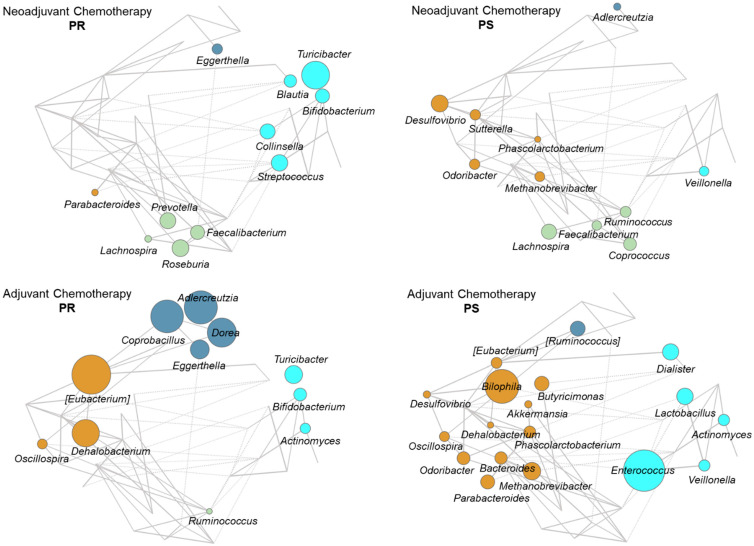
Co-abundance groups in the gut microbiota of EOC patients receiving neoadjuvant or adjuvant chemotherapy. Wiggum plots show the pattern of variation of the four identified co-abundance groups (CAGs) in EOC patients receiving neoadjuvant (top) or adjuvant (bottom) chemotherapy. Patients were stratified by response to therapy: PR, platinum-resistant (left) vs. PS, platinum-sensitive (right). CAGs were named according to the most abundant genera and color coded as follows: *Bacteroides* (brown), *Collinsella* (cyan), *[Ruminococcus]* (dark blue), and *Faecalibacterium* (green). Each genus is depicted as a node whose size is proportional to the over-abundance relative to background. Positive and significant Kendall correlations between two or more genera are indicated with lines connecting the nodes (*p* < 0.05). The thickness of the lines was drawn in proportion to the correlation strength. See also Figure S8.

To further explore the GM compositional variation in relation to the therapeutic response, we established co-abundance associations of genera and then clustered correlated taxa into co-abundance groups (CAGs) describing the GM structures found across the whole dataset (permutation multivariate analysis of variance, *p* < 0.05; see Figure S8). Four CAGs were identified and named based on the dominant (i.e., the most abundant) genus within each of them (*Bacteroides*, *Collinsella*, *[Ruminococcus]*, and *Faecalibacterium*). The CAG relationships are shown as Wiggum plots in Figure 6. Regardless of the treatment group, PR patients were characterized by *Collinsella* CAG (cyan), especially the co-abundance of *Bifidobacterium* and *Turicibacter*, as well as by *Eggerthella*, belonging to the *[Ruminococcus]* CAG (dark blue). The *Collinsella* CAG was also represented in PS patients but with mostly distinct genera, including *Veillonellaceae* members. On the other hand, PS patients shared an overrepresentation of the *Bacteroides* CAG (brown) with co-abundance of *Desulfovibrio*, *Odoribacter*, *Phascolarctobacterium*, and *Methanobrevibacter* along with other treatment group-specific genera. Again, the *Bacteroides* CAG was also represented in PR patients, especially those receiving adjuvant therapy, but with much increased proportions of *[Eubacterium]* and *Dehalobacterium*. The *Faecalibacterium* CAG (green) with co-abundance of well-known SCFA producers such as *Faecalibacterium*, *Lachnospira*, *Ruminococcus*, *Roseburia*, and *Coprococcus* was mostly represented in the neoadjuvant treatment group regardless of response.

### 3.5. Predicted Functional Profiling of the Gut Microbiota in EOC Patients during Chemotherapy

**Figure 7:**
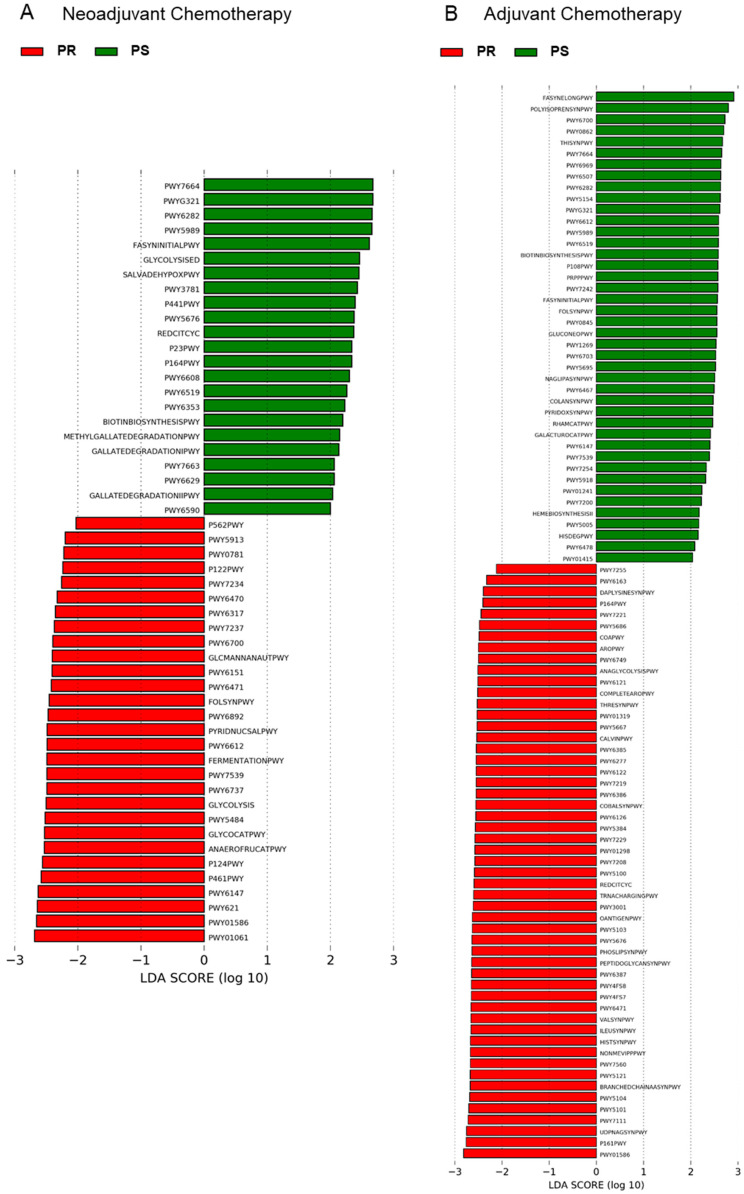
Potential inferred metagenomics signatures of therapeutic response in EOC patients receiving neoadjuvant or adjuvant chemotherapy. Differentially represented inferred functions between platinum-sensitive (i.e., PS) and platinum-resistant (i.e., PR) EOC patients receiving neoadjuvant (**A**) or adjuvant (**B**) chemotherapy were identified by linear discriminant analysis (LDA) effect size (LEfSe) analysis. The logarithmic threshold for discriminative features was set to 2.0. See also Table S1 for MetaCyc pathways and corresponding superclasses.

In order to gain insights into the functional variation of the GM of EOC patients during chemotherapy, 16S rRNA gene sequencing data were used to predict GM functionalities through the PICRUSt2 pipeline [26]. A total of 129 MetaCyc pathways were identified by LEfSe as potential functional markers of response (Figure 7 and Table S1). In particular, in the adjuvant group, almost all of the discriminating functionalities (83.0% for PR patients and 73.2% for PS patients) belonged to the class of biosynthesis, i.e., they were involved in the synthesis of small molecules, macromolecules, and cell structure components (e.g., nucleotides, amino acids, vitamins, carbohydrates, fatty acids, and lipids). These biosynthetic capacities were also similarly represented between PR and PS patients from the neoadjuvant group but with lower overall values (37.9% for PR and 39.0% for PS patients). The rest of the discriminating functionalities were associated with the degradation of the same molecules or with the generation of energy precursors. Interestingly, among the predicted functions that discriminated for PR patients, we found pathways directly involved in fermentation to lactate (i.e., homolactic fermentation, heterolactic fermentation, mixed acid fermentation, *Bifidobacterium* shunt, hexitol fermentation to lactate, formate, ethanol, and acetate in the neoadjuvant group and pyruvate fermentation to acetate and lactate II in the adjuvant group). In contrast, none of the inferred GM activities characteristic of PS patients were associated with lactate production for any of the chemotherapy regimens.

## 4. Discussion

As far as we know, for the first time, we performed a prospective study on the profile of GM of women with EOC and reconstructed its trajectory over the course of chemotherapy in relation to disease severity (and consequently to the treatment administered, neoadjuvant vs. adjuvant) and therapeutic response (PR vs. PS). As already observed in other tumor contexts [40,41,42,43], the GM of patients at baseline (i.e., before anti-cancer treatments) showed some unbalanced, potentially dysbiotic traits, compared to healthy subjects matched for several GM-associated confounding factors (i.e., age, sex, BMI, and geography). In particular, EOC patients were depleted of most of the typically health-associated GM members, such as *Blautia*, *Coprococcus*, *Lachnospira*, *Dorea*, and *Roseburia*, all of which belong to the *Lachnospiraceae* family, as well as *Bifidobacterium*, a well-known commensal probiotic. It is widely recognized that these microorganisms produce SCFAs, i.e., key metabolites to maintain host metabolic, immunological, and neurological homeostasis [44,45], and their decrease probably represents a non-specific shared response to diseases, as recently discussed [46]. On the other hand, EOC patients were particularly enriched in members of *Coriobacteriaceae*, including *Adlercreutzia* and especially *Collinsella*. As for the latter, it was recently associated with metabolic disorders [47,48] and suggested to play a role in altering intestinal permeability, contributing to a pro-inflammatory state through increased production of chemokines and IL-17A, even in other pathological contexts, such as rheumatoid arthritis [49]. It is also worth noting that *Collinsella* as well as other *Coriobacteriaceae* components were found to be overrepresented in the GM of some cancer patients, including those with higher grade breast and colorectal cancer [50,51]. Although we are still a long way from defining “healthy” vs. “dysbiotic” microbiomes [52], all these characteristics could be associated with loss of intestinal homeostasis that, with adequate caution, could be linked to the development and/or the progression of various disorders.

In line with the literature available on the effects of systemic anticancer therapy on GM in different types of cancer, including different chemotherapy agents and treatment settings [53], we found that chemotherapy further aggravated the GM imbalance of EOC patients with reduction of alpha diversity, further decline in the proportions of beneficial microbes, such as *Lachnospiraceae* and *Ruminococcaeae* members, and a further increase in *Coriobacteriaceae*. However, the most striking findings emerged when the trajectories of GM were re-analyzed in relation to the therapeutic response. In fact, we observed that PR and PS patients underwent distinct dynamics of alpha and beta diversity and differed in the relative abundance trends of certain taxa. Specifically, a progressive reduction of alpha diversity was observed for PR patients, while in PS patients, the diversity values remained mostly stable and generally higher. Higher values of alpha diversity halfway through the chemotherapy treatment were associated with longer progression-free and overall survival in the whole cohort. Moreover, unlike PS patients, the GM of PR patients underwent considerable fluctuations in terms of beta diversity mostly higher than the baseline inter-patient variability, thus highlighting a high degree of intra-patient temporal instability. It should be remembered that diversity and stability are essential ecological characteristics of GM [54] and are considered hallmarks of a healthy intestine and generally good health, the loss of which is repeatedly associated with poor prognosis ranging from infectious complications to mortality [35,55]. From a taxonomic standpoint, the GM of PR patients preserved the overabundance of *Coriobacteriaceae* members—particularly *Eggerthella*—and exhibited a peculiar overrepresentation of *Bifidobacterium* over time regardless of chemotherapy treatment group. It is worth noting that all of these microorganisms produce lactate as part of their metabolism [49,56,57], as also supported by inferred metagenomics. Furthermore, *Eggerthella* is known to use arginine as an energy source with the production of ornithine [58], which in turn serves as a precursor for the biosynthesis of putrescine, spermidine, and spermine. In light of the established role of polyamines in multiple oncogenic pathways [59], it is possible to speculate that the extra supply of precursors by *Eggerthella* might replenish the polyamine pool, thus contributing to tumor progression and therefore to therapy failure. As for *Bifidobacterium*, its overrepresentation in the GM of PR patients apparently contrasts with the data available in the literature that indicate it as an immunotherapy-favorable microorganism [16,60]. However, some strains were also shown to promote immunotolerance via T-regulatory cells [61], which may be unfavorable in some cancer settings, such as EOC [62]. On the other hand, *Veillonella, Megasphaera* and *Dialister*, all lactate utilizers belonging to the *Veillonellaceae* family, along with the SCFA producers *Catenibacterium* and *Anaerotruncus* were distinctly associated with a more favorable platinum response (i.e., they were overabundant in PS patients). Lactate is a key element in tumor metabolism. Produced in copious amounts by cancer cells through increased aerobic glycolysis (the so-called Warburg effect), it is necessary for their self-sufficiency; it stimulates angiogenesis and tumor growth, promotes cell migration and metastasis, and contributes to immune escape by acidification of the tumor microenvironment [63,64]. On the other hand, as discussed above, lactate is also a common fermentation product of some GM members, although it is generally not detectable in feces, as it is absorbed by intestinal cells or used by lactate-consuming taxa. Among the latter, *Veillonella* was recently implicated in the disposal of exercise-induced lactate, which can cross to the gut lumen, thus becoming available for alternative catabolic routes [65]. It is therefore tempting to hypothesize that the aforementioned GM signatures of therapeutic response (i.e., increased representation of lactate-utilizing bacteria and reduced proportions of lactate producers and ability to ferment to lactate) are linked to the lactate cycle, altering its bioavailability and thus influencing tumor progression and the efficacy of chemotherapy. It must be said that none of the discriminating microorganisms identified were differentially represented at the time of diagnosis between PR and PS patients, suggesting the existence of a critical time window during the chemotherapy treatment in which peculiar microbial networks are established that could influence the efficacy of therapies. Not least, some potential microbial signatures of therapeutic failure appeared in the GM of PS patients of the neoadjuvant group in the follow-up (i.e., *Adlercreutzia* and *Eggerthella* overabundances) parallel to the onset of relapses, which further strengthens their relevance as possible prognostic markers. Despite the interesting results of this work, several limitations should be mentioned: the small sample size (especially the low number of PR patients receiving adjuvant chemotherapy), the inclusion of a single histotype (which limits the generalizability of our findings), the unsystematic sampling (for practical reasons, fecal samples were not collected at every time-point), and the inability to measure circulating lactate levels. Furthermore, although high-grade serous EOC is often associated with pelvic masses compressing the sigmoid and the rectum, thus leading to constipation and subocclusion, we did not systematically collect information on bowel movement quality, which is known to be among the main microbiota-associated confounding factors [38].

## 5. Conclusions

This pilot study highlighted distinct GM trajectories in EOC patients during chemotherapy treatment closely related to the therapeutic outcome. In particular, the potential GM signatures found suggest the possible involvement of certain GM members in regulating the levels of the oncometabolite lactate through enhanced production (lactagenesis) or disposal. This new evidence related to the GM-EOC relationship needs to be confirmed in larger cohort studies involving multiple histological types with different chemosensitivity and prognosis, collection of various biological samples (including blood) and host metadata, and possibly the combination of different approaches, including omics, functional analysis, and animal models, to uncover the role of intestinal microorganisms in favoring or, vice versa, worsening the clinical response to EOC therapy. Correlation vs. causation needs to be dissected as well.

## Figures and Tables

**Table 1:** Patients’ characteristics.

Patient ID	Therapeutic Program	Age (Years)	BMI (kg/m^2^)	FIGO Stage	BRCA Gene Status	Nr. Cycles	PCI	CA125 at Diagnosis (U/mL)	Residual Disease (CC)	PFS (Months)	OS (Months)	Follow-Up State	Response to Platinum
MiCrO 1	neoadj	51	20	IV	WT	15	16	1030	CC-2	1	9	DWD	PR
MiCrO 2	neoadj	68	34	IIIC	WT	6	22	1800	CC-0	10	21	DWD	PR
MiCrO 4	neoadj	71	25	III	WT	9	16	1256	CC-0	24	39	AWD	PS
MiCrO 6	neoadj	71	21	IIIC	WT	10	20	1200	CC-1	14	21	DWD	PR
MiCrO 8	neoadj	48	21	III	BRCA1m	6	18	999	CC-0	15	16	AWD	PS
MiCrO 9	neoadj	58	24	IV	WT	8	25	1100	CC-0	29	30	NED	PS
MiCrO 10	neoadj	56	20	IIIC	WT	9	27	2100	CC-0	12	17	DWD	PR
MiCrO 16	neoadj	55	21	III	BRCA2m	6	25	1050	CC-0	23	23	AWD	PS
MiCrO 23	neoadj	51	20	IIIC	BRCA1m	9	21	499	CC-0	18	25	AWD	PS
MiCrO 24	neoadj	61	21	III	WT	13	24	499	CC-0	13	13	NED	PS
MiCrO 25	neoadj	67	20	IIIC	WT	9	27	999	CC-2	16	22	AWD	PR
MiCrO 28	neoadj	48	24	IIIC	BRCA1m	6	37	1030	CC-0	12	12	DWD	PR
MiCrO 3	adj	69	23	IIIC	WT	6	18	1000	CC-0	8	8	NED	PS
MiCrO 5	adj	61	23	IIIC	WT	6	17	750	CC-1	14	21	AWD	PS
MiCrO 7	adj	57	21	IV	WT	6	29	1029	CC-1	10	13	DWD	PR
MiCrO 11	adj	67	23	IIIC	BRCA1m	6	21	1340	CC-0	11	18	AWD	PR
MiCrO 12	adj	43	22	IIB	WT	6	3	1000	CC-0	13	13	NED	PS
MiCrO 13	adj	53	23	IV	WT	8	20	850	CC-2	14	14	AWD	PR
MiCrO 17	adj	53	19	IV	BRCA1m	6	18	1000	CC-0	17	18	AWD	PS
MiCrO 19	adj	57	20	IC	BRCA1/2m	6	0	679	CC-0	15	15	NED	PS
MiCrO 22	adj	39	20	IV	BRCA1m	6	30	840	CC-0	18	18	AWD	PS
MiCrO 31	adj	68	24	IIIB	BRCA1m	6	3	350	CC-0	15	15	AWD	PS
MiCrO 33	adj	69	25	IIIC	WT	6	22	286	CC-0	12	12	NED	PS
MiCrO 39	adj	54	32	IIIC	WT	6	11	453	CC-0	10	10	NED	PS

Adj = adjuvant chemotherapy; AWD = alive with disease; BMI = body mass index; BRCAm = breast cancer gene mutation; CC = completeness cytoreduction; DWD = death with disease; FIGO = International Federation of Obstetrics and Gynecology; NED = no evidence of disease; Neoadj = neoadjuvant chemotherapy; OS = overall survival; PCI = peritoneal cancer index; PFS = progression-free survival; PR = platinum-resistant; PS = platinum-sensitive; WT = wild-type.

## Data Availability

Raw sequence reads of 16S rRNA gene sequencing were deposited in the National Center for Biotechnology Information Sequence Read Archive (NCBI SRA, BioProject: PRJNA726917).

## Supplementary Materials

The following are available online at https://www.mdpi.com/article/10.3390/cancers13163999/s1, Figure S1: Alpha and beta diversity in EOC patients during neoadjuvant or adjuvant chemotherapy, Figure S2: The compositional structure of the gut microbiota of EOC patients during neoadjuvant or adjuvant chemotherapy, Figure S3: Beta diversity in EOC patients stratified by therapeutic response during neoadjuvant and adjuvant chemotherapy, Figure S4: The gut microbiota of EOC patients at diagnosis does not stratify by disease severity or therapeutic response, Figure S5: Temporal dynamics of the main discriminating taxa for platinum-resistant or platinum-sensitive EOC patients during neoadjuvant or adjuvant chemotherapy, Figure S6: Association of gut microbial taxa and survival in EOC patients receiving chemotherapy treatments, Figure S7: Variations in the potential gut microbial signatures of therapeutic response in the follow-up, up to 6 months after the end of therapy, Figure S8: Assignment of bacterial co-abundance groups (CAGs), Table S1: MetaCyc pathway and superclass information for the discriminating pathways between platinum-resistant and platinum-sensitive EOC patients receiving neoadjuvant or adjuvant chemotherapy.
