# Supplementary material for: Gut Microbiota Dynamics during Chemotherapy in Epithelial Ovarian Cancer Patients Are Related to Therapeutic Outcome
Source: Cancers (Basel). 2021 Aug 8;13(16):3999. doi: 10.3390/cancers13163999 (PMC8393652; doi:10.3390/cancers13163999)
Supplement: Supplementary file 1 [file cancers-13-03999-s001.zip › cancers-1264786-supplementary.pdf]

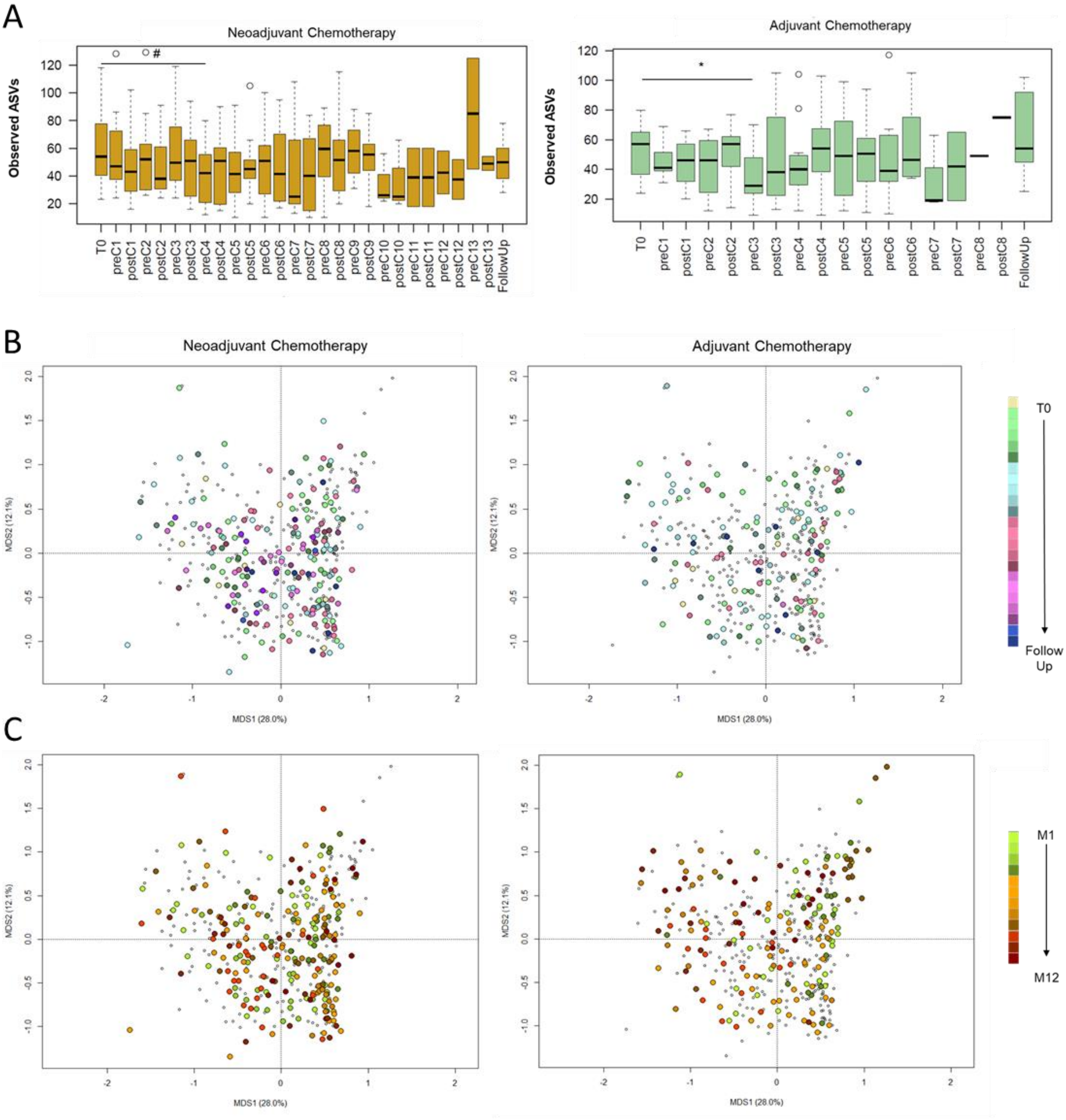


**Figure S1. Alpha and beta diversity in EOC patients during neoadjuvant or adjuvant chemotherapy.**

**A**, Boxplots showing the distribution of the number of observed ASVs during neoadjuvant (left) or adjuvant (right) chemotherapy (Wilcoxon test, * for $p \leq 0.05$; # for $0.05 < p \leq 0.1$). C, chemotherapy cycle.

PCoA based on weighted UniFrac distances between the gut microbiota profiles of EOC patients receiving neoadjuvant (left) or adjuvant (right) chemotherapy, stratified by collection time (from T0 until follow-up, **B**) or patient (from M1 to M12, **C**). A significant separation by patient was found (permutation test with pseudo-F ratio, $p = 0.001$).

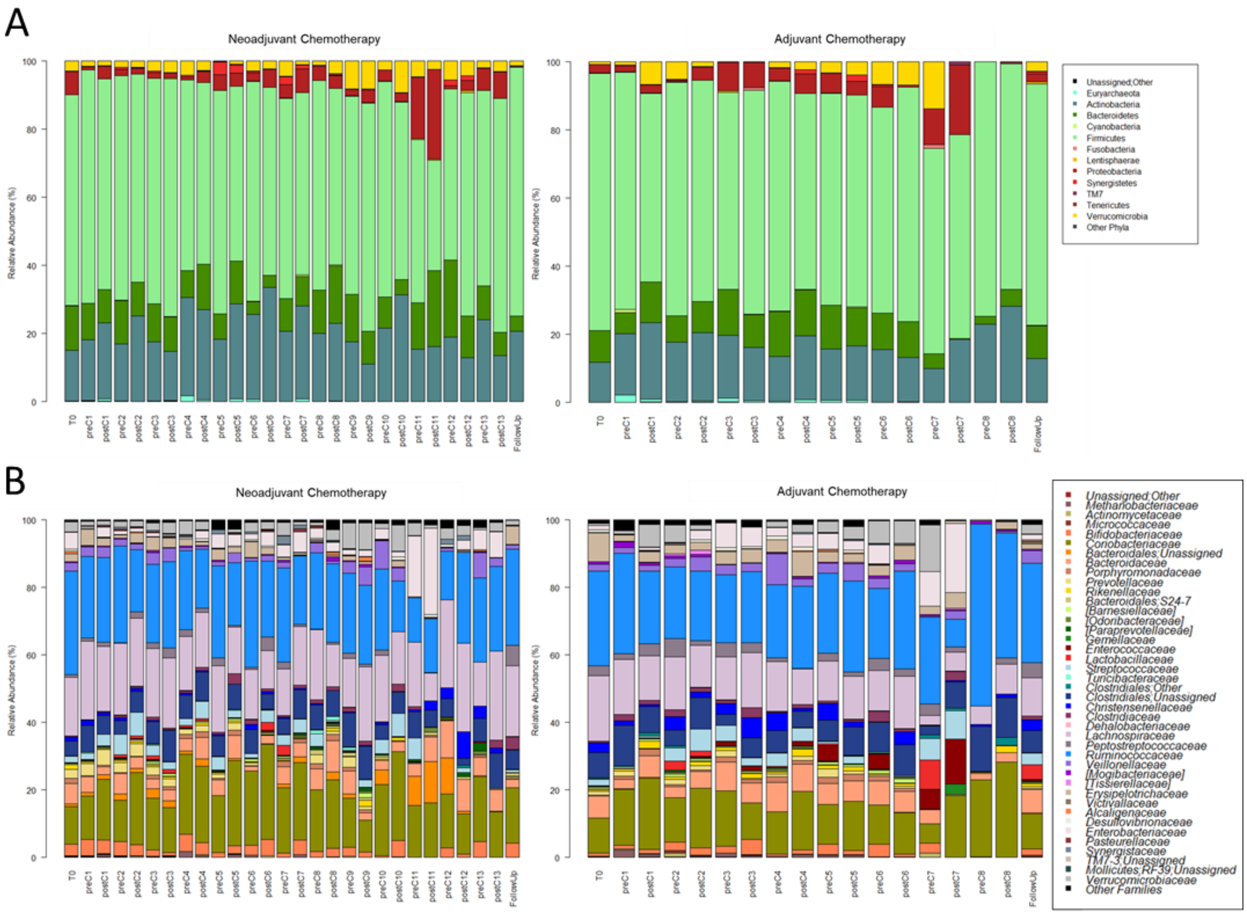


**Figure S2. The compositional structure of the gut microbiota of EOC patients during neoadjuvant or adjuvant chemotherapy.**

Bar plots showing the relative abundance of phyla (**A**) and families (**B**) of the gut microbiota of EOC patients during neoadjuvant (left) or adjuvant (right) chemotherapy. C, chemotherapy cycle. Only taxa with relative abundance > 0.1% in at least one sample are shown.

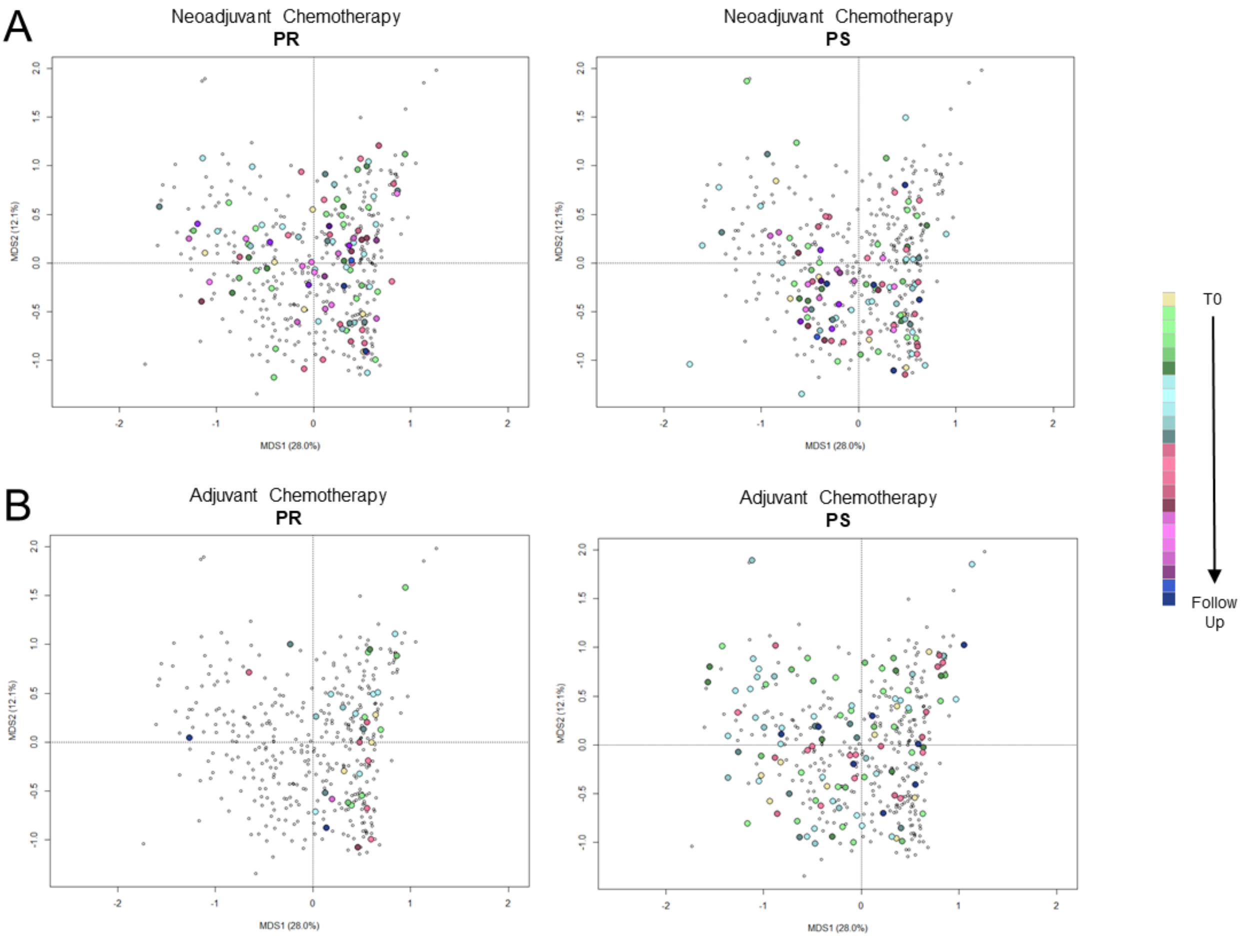


**Figure S3. Beta diversity in EOC patients stratified by therapeutic response, during neoadjuvant and adjuvant chemotherapy.**

PCoA based on weighted UniFrac distances between the gut microbiota profiles of platinum-resistant (*i.e.*, PR, left) and platinum-sensitive (*i.e.*, PS, right) EOC patients during neoadjuvant (**A**) or adjuvant (**B**) chemotherapy, stratified by collection time (from T0 until follow-up). No significant separation was found (permutation test with pseudo-F ratio, $p > 0.05$).

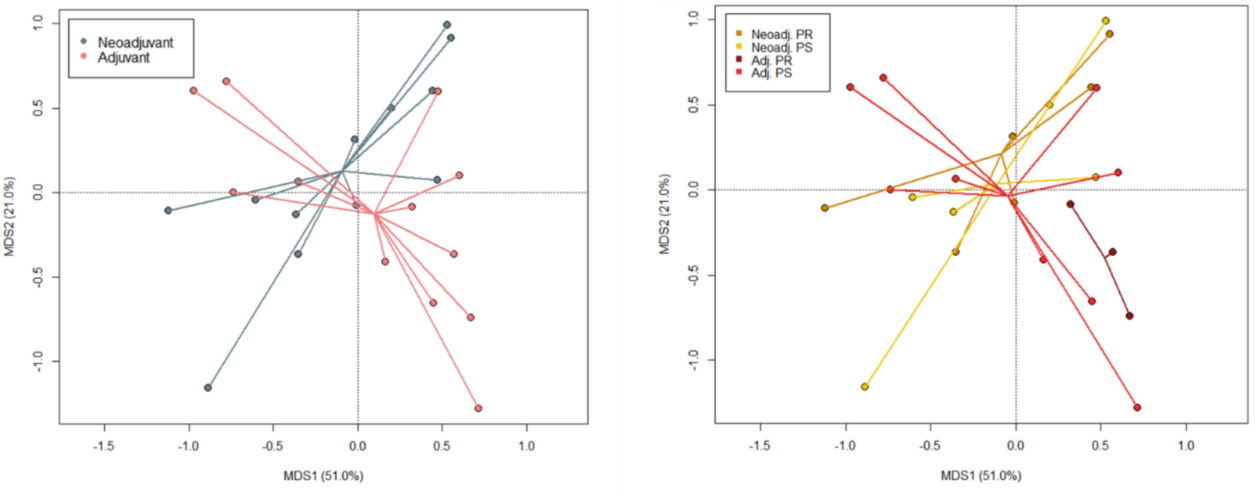


**Figure S4. The gut microbiota of EOC patients at diagnosis does not stratify by disease severity or therapeutic response.**

PCoA based on weighted UniFrac distances between the gut microbiota profiles of EOC patients at diagnosis, stratified by disease severity and consequently treatment administered (*i.e.*, neoadjuvant vs. adjuvant chemotherapy, left), or therapeutic response (PR, platinum-resistant vs. PS, platinum-sensitive for each treatment group, right). No significant separation was found (permutation test with pseudo-F ratio, $p > 0.05$).

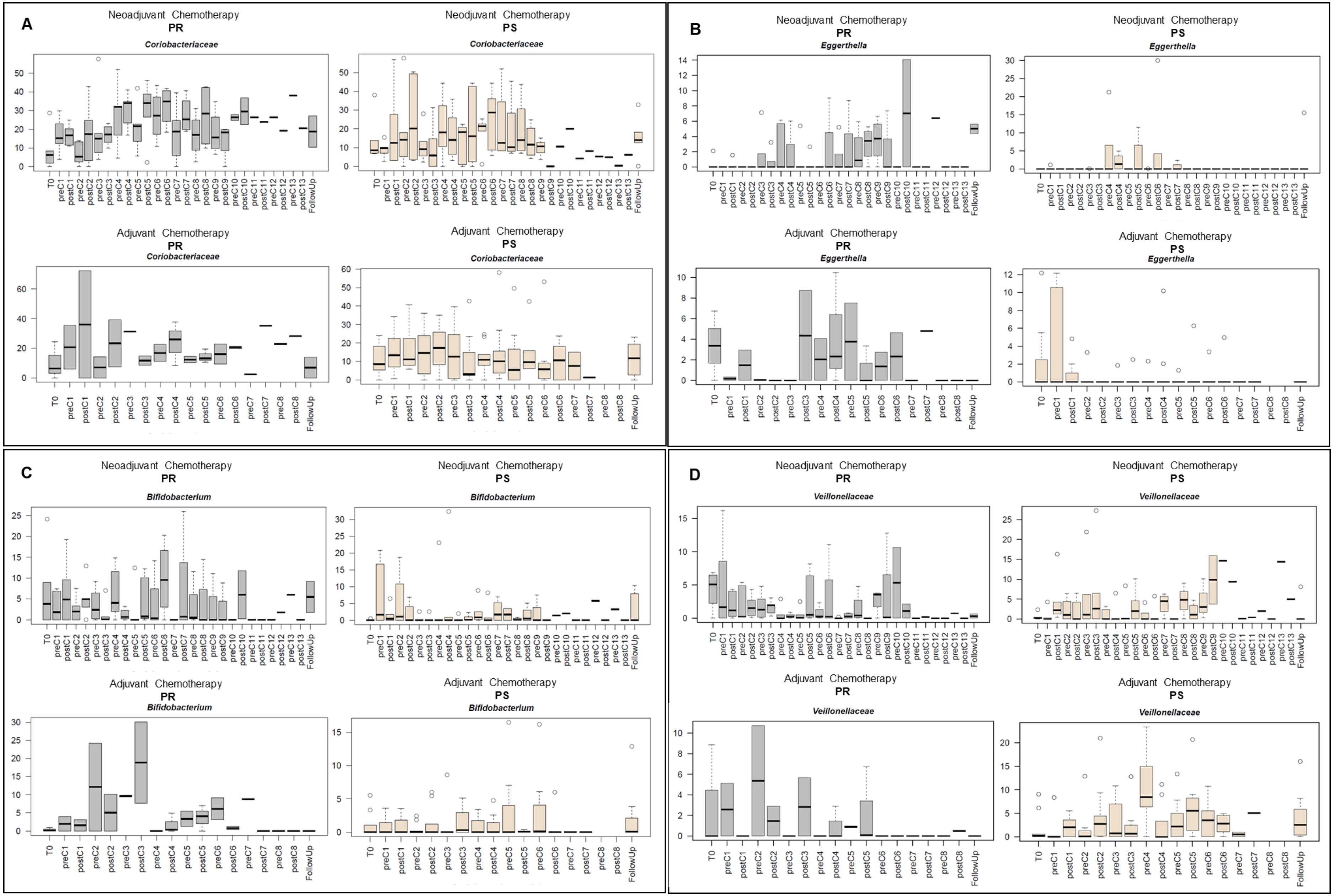
A
Neoadjuvant Chemotherapy
PR
Coriobacteriaceae
Neodjuvant Chemotherapy
PS
Coriobacteriaceae
Adjuvant Chemotherapy
PR
Coriobacteriaceae
Adjuvant Chemotherapy
PS
Coriobacteriaceae
B
Neoadjuvant Chemotherapy
PR
Eggerthella
Neodjuvant Chemotherapy
PS
Eggerthella
Adjuvant Chemotherapy
PR
Eggerthella
Adjuvant Chemotherapy
PS
Eggerthella
C
Neoadjuvant Chemotherapy
PR
Bifidobacterium
Neodjuvant Chemotherapy
PS
Bifidobacterium
Adjuvant Chemotherapy
PR
Bifidobacterium
Adjuvant Chemotherapy
PS
Bifidobacterium
D
Neoadjuvant Chemotherapy
PR
Veillonellaceae
Neodjuvant Chemotherapy
PS
Veillonellaceae
Adjuvant Chemotherapy
PR
Veillonellaceae
Adjuvant Chemotherapy
PS
Veillonellaceae

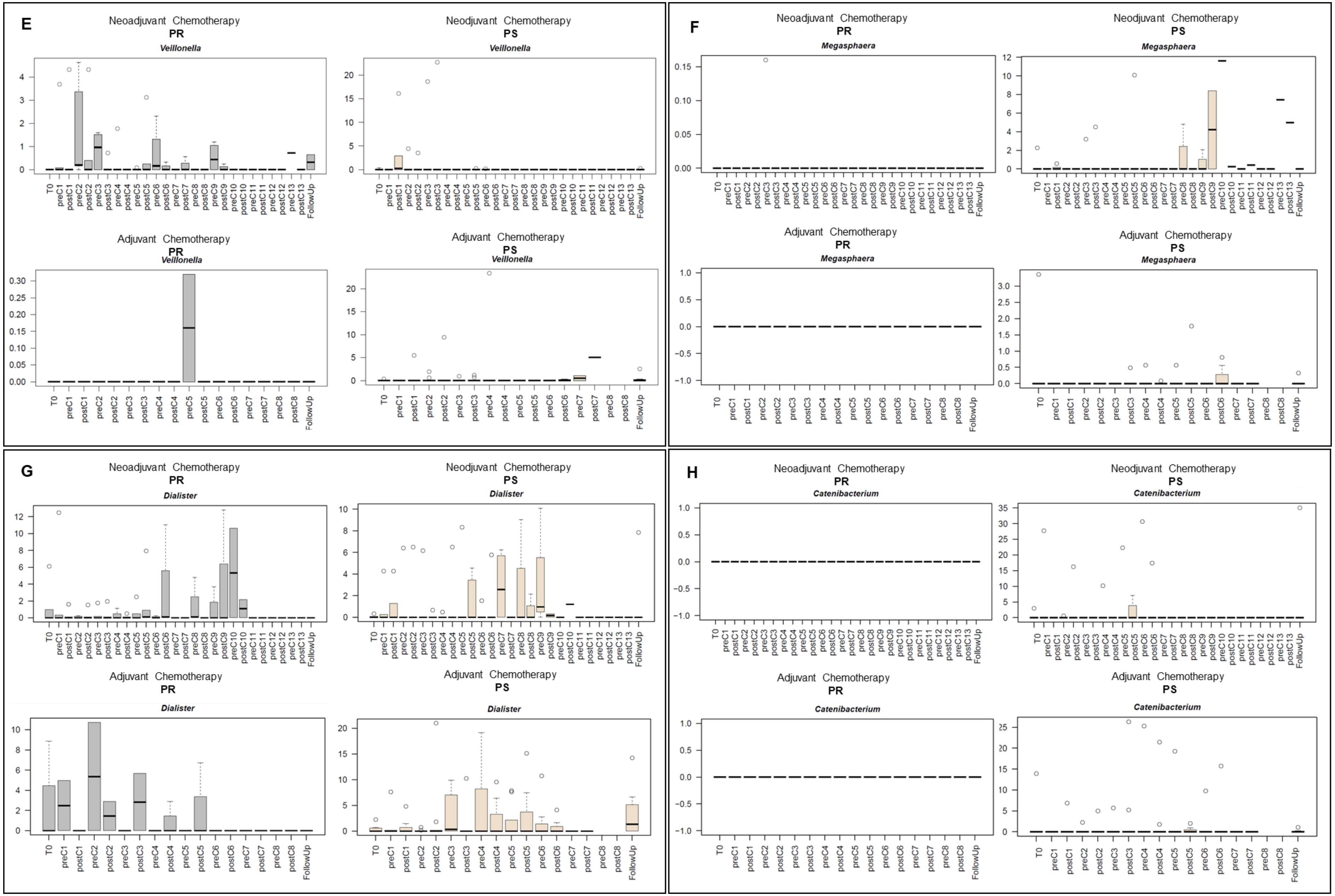
E
Neoadjuvant Chemotherapy
PR
Veillonella
Neodjuvant Chemotherapy
PS
Veillonella
Adjuvant Chemotherapy
PR
Veillonella
Adjuvant Chemotherapy
PS
Veillonella
F
Neoadjuvant Chemotherapy
PR
Megasphaera
Neodjuvant Chemotherapy
PS
Megasphaera
Adjuvant Chemotherapy
PR
Megasphaera
Adjuvant Chemotherapy
PS
Megasphaera
G
Neoadjuvant Chemotherapy
PR
Dialister
Neodjuvant Chemotherapy
PS
Dialister
Adjuvant Chemotherapy
PR
Dialister
Adjuvant Chemotherapy
PS
Dialister
H
Neoadjuvant Chemotherapy
PR
Catenibacterium
Neodjuvant Chemotherapy
PS
Catenibacterium
Adjuvant Chemotherapy
PR
Catenibacterium
Adjuvant Chemotherapy
PS
Catenibacterium
T0
preC1
postC1
preC2
postC2
preC3
postC3
preC4
postC4
preC5
postC5
preC6
postC6
preC7
postC7
preC8
postC8
preC9
postC9
preC10
postC10
preC11
postC11
preC12
postC12
preC13
postC13
FollowUp

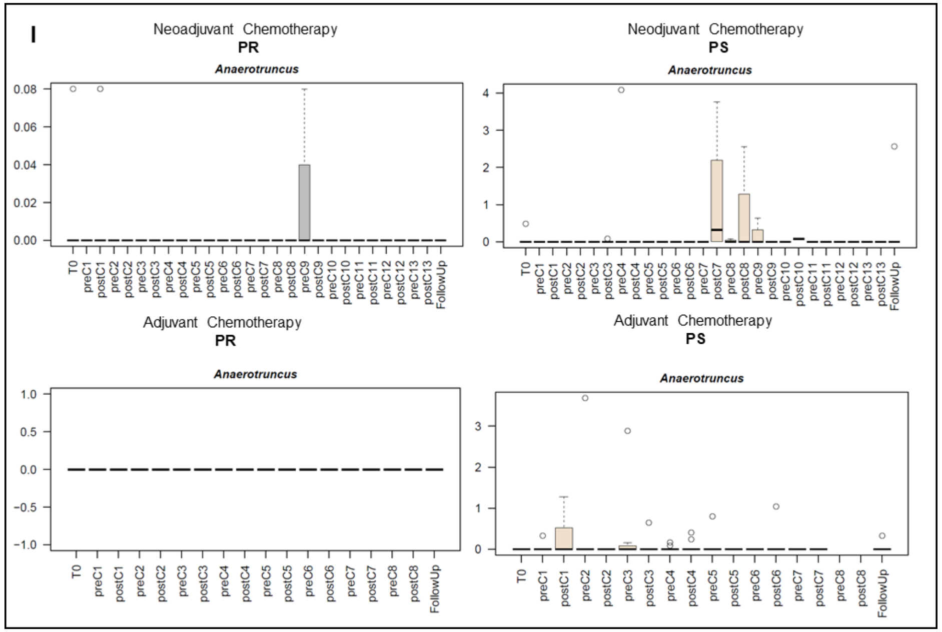


**Figure S5. Temporal dynamics of the main discriminating taxa for platinum-resistant or platinum-sensitive EOC patients during neoadjuvant or adjuvant chemotherapy.**

Boxplots showing the relative abundance distribution of the main taxa identified as discriminating for platinum-resistant (PR) or platinum-sensitive (PS) EOC patients, based on LEfSe analysis, during neoadjuvant or adjuvant chemotherapy. The following families and genera are shown: **A**, *Coriobacteriaceae*; **B**, *Eggerthella*; **C**, *Bifidobacterium*; **D**, *Veillonellaceae*; **E**, *Veillonella*; **F**, *Megasphaera*; **G**, *Dialister*; **H**, *Catenibacterium*; **I**, *Anaerotruncus*. For each panel, boxplots are shown in the following order: top, neoadjuvant group vs. bottom, adjuvant group; left, PR patients vs right, PS patients. C, chemotherapy cycle. See also **Figure 5**.

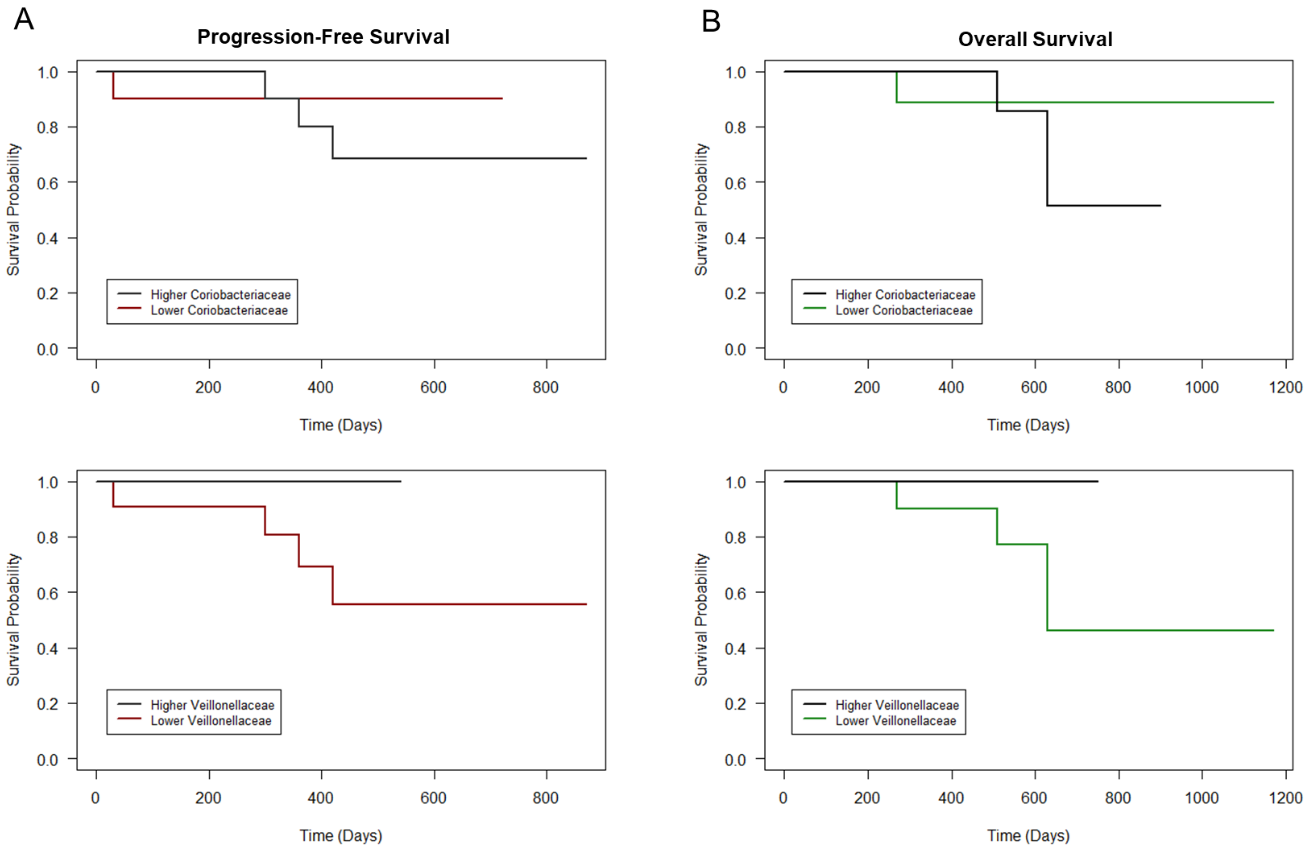


**Figure S6. Association of gut microbial taxa and survival in EOC patients receiving chemotherapy treatments.**

Kaplan-Meier curves for progression-free survival (**A**) and overall survival (**B**) in the whole cohort. EOC patients were stratified by higher or lower relative abundance of *Coriobacteriaceae* (top) and *Veillonellaceae* (bottom) (relative to median) at the preC7 (for neoadjuvant therapy) or preC4 (for adjuvant therapy) time-point. Log-rank test, $p \leq 0.1$ for *Veillonellaceae*.

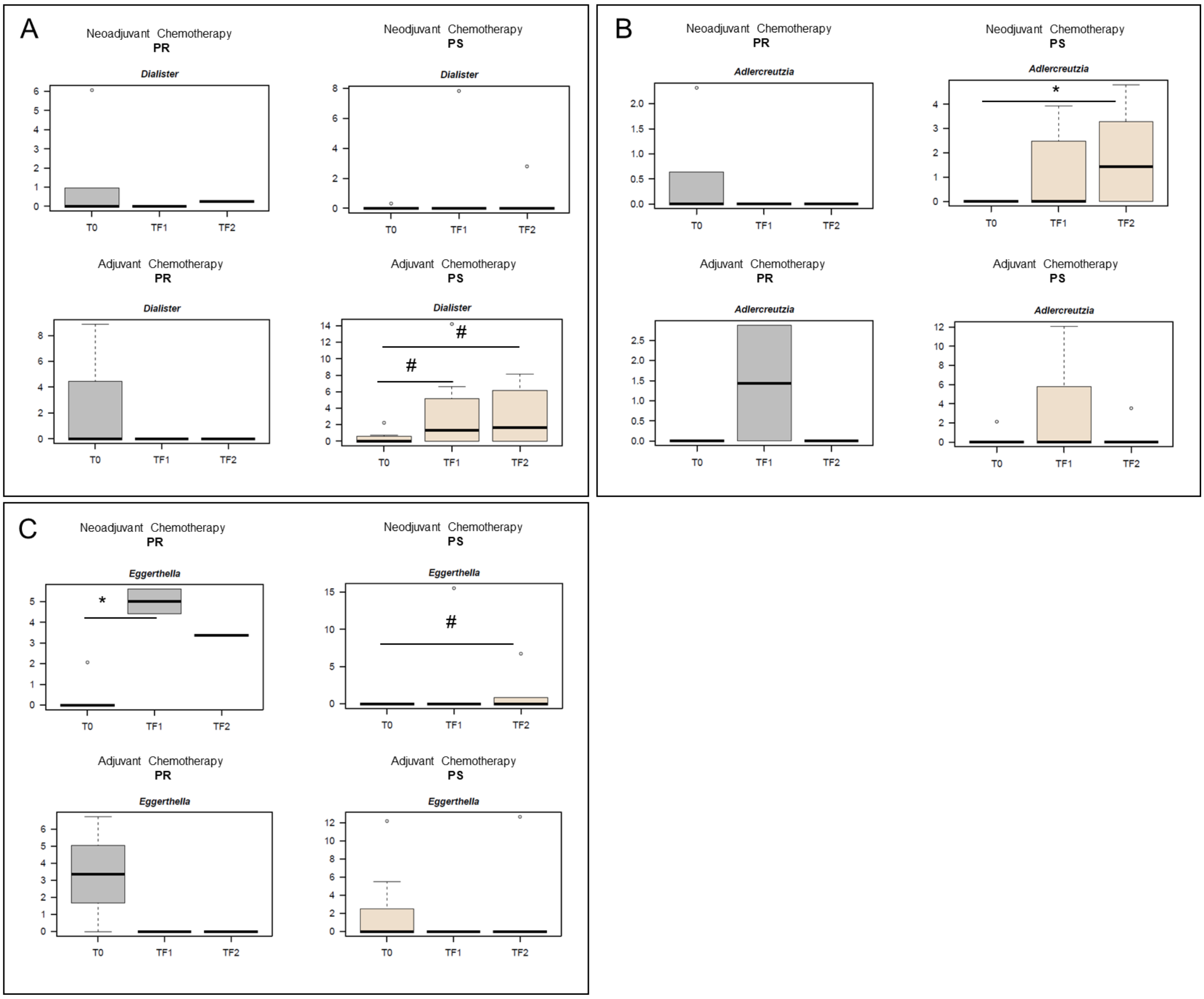


**Figure S7. Variations in the potential gut microbial signatures of therapeutic response in the follow-up, up to 6 months after the end of therapy.**

Boxplots showing the relative abundance distribution of discriminating taxa for platinum-resistant (PR) or platinum-sensitive (PS) EOC patients, whose proportions remained high or increased in the follow-ups, at 3 (TF1) and 6 (TF2) months from the end of therapy, compared to baseline (T0). Discriminating taxa were identified by LEfSe (see **Figure 5**). The following genera are shown: **A**, *Dialister*; **B**, *Adlercreutzia*; **C**, *Eggerthella*. For each panel, boxplots are shown in the following order: top, neoadjuvant group vs. bottom, adjuvant group; left, PR patients vs right, PS patients. Wilcoxon test, * for $p \leq 0.05$; $^{\#}$ for $0.05 < p \leq 0.\ 1$.

A

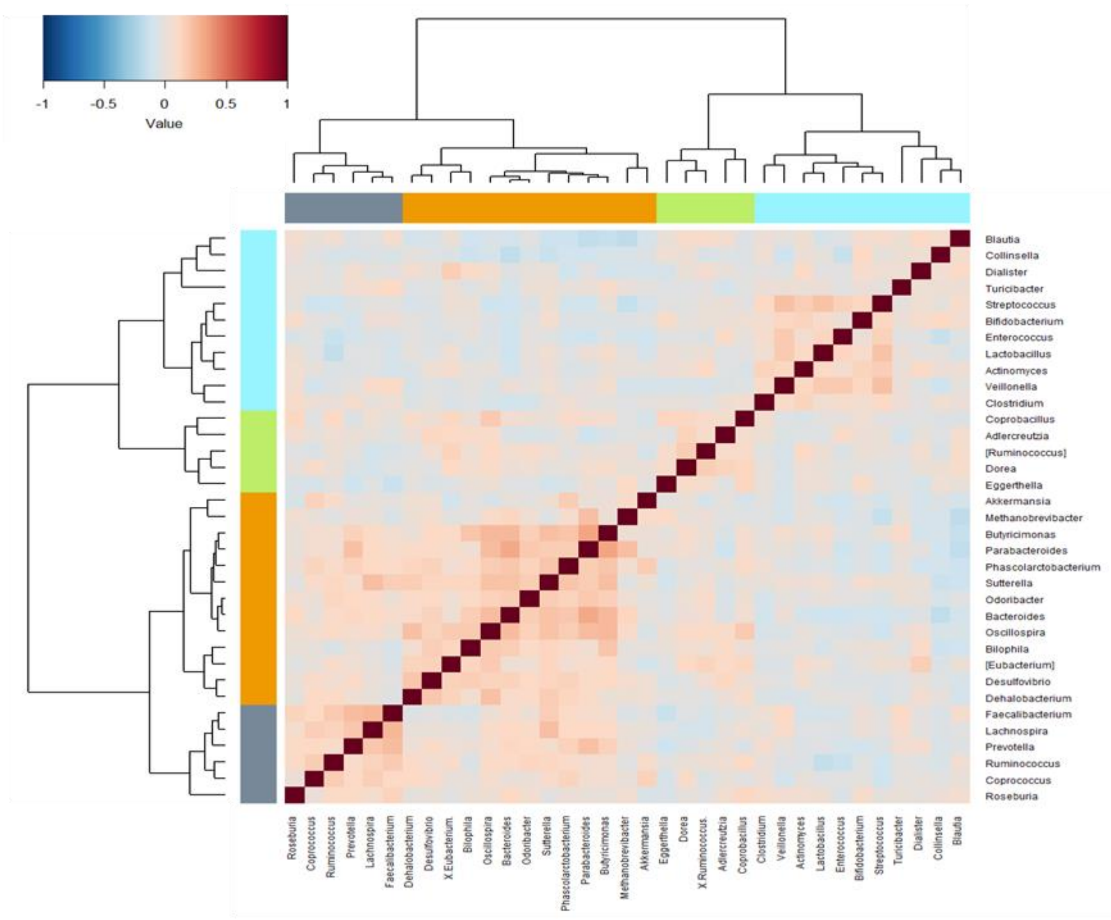
-1
-0.5
0
0.5
1
Value
Blautia
Collinsella
Dialister
Turicibacter
Streptococcus
Bifidobacterium
Enterococcus
Lactobacillus
Actinomyces
Veillonella
Clostridium
Coprobacillus
Adlercreutzia
[Ruminococcus]
Dorea
Eggerthella
Akkermansia
Methanobrevibacter
Butyricimonas
Parabacteroides
Phascolarctobacterium
Sutterella
Odoribacter
Bacteroides
Oscillospira
Bilophila
[Eubacterium]
Desulfovibrio
Dehalobacterium
Faecalibacterium
Lachnospira
Prevotella
Ruminococcus
Coprococcus
Roseburia
X.Eubacterium.
X.Ruminococcus.

B

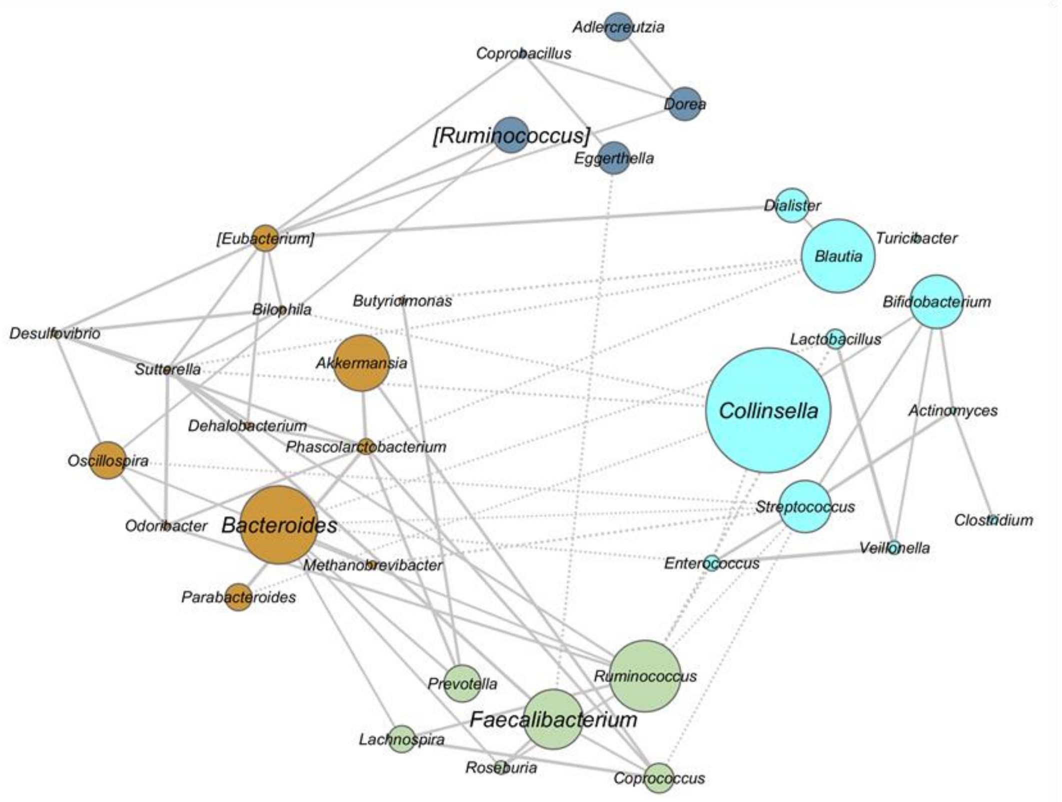
Adlercreutzia
Coprobacillus
Dorea
[Ruminococcus]
Eggerthella
Dialister
[Eubacterium]
Blautia
Turicibacter
Butyricimonas
Bifidobacterium
Bilophila
Desulfovibrio
Lactobacillus
Sutterella
Akkermansia
Collinsella
Actinomyces
Dehalobacterium
Phascolarctobacterium
Oscillospira
Streptococcus
Clostridium
Odoribacter
Bacteroides
Veillonella
Methanobrevibacter
Enterococcus
Parabacteroides
Ruminococcus
Prevotella
Faecalibacterium
Lachnospira
Roseburia
Coprococcus

**Figure S8. Assignment of bacterial co-abundance groups (CAGs).**

**A**, Heat plot showing Kendall correlations between genera clustered by Spearman correlation coefficient and Ward linkage. The colors are indicative of the four CAGs identified.

**B**, Wiggum plot showing correlations between CAGs. The circle size is proportional to the genus abundance and connections between nodes represent positive and significant Kendall correlations between genera ($p < 0.05$).

| MetaCyc ID | Pathway | Superclasses I | Superclasses II | Superclasses III |
|---|---|---|---|---|
| FASYN-ELONG-PWY | fatty acid elongation -- saturated | Biosynthesis → Fatty Acid and Lipid Biosynthesis → Fatty Acid Biosynthesis | | |
| POLYISOPRENSYN-PWY | polyisoprenoid biosynthesis (E. coli) | Biosynthesis → Polyprenyl Biosynthesis → All-trans Polyprenyl Biosynthesis | Superpathways | |
| PWY-6700 | queuosine biosynthesis I (de novo) | Macromolecule Modification → Nucleic Acid Processing → Queuosine Biosynthesis and Salvage | | |
| PWY0-862 | (5Z)-dodecenoate biosynthesis I | Biosynthesis → Fatty Acid and Lipid Biosynthesis → Fatty Acid Biosynthesis → Unsaturated Fatty Acid Biosynthesis | | |
| THISYN-PWY | superpathway of thiamine diphosphate biosynthesis I | Biosynthesis → Cofactor, Carrier, and Vitamin Biosynthesis → Enzyme Cofactor Biosynthesis → Thiamine Biosynthesis | Biosynthesis → Cofactor, Carrier, and Vitamin Biosynthesis → Vitamin Biosynthesis → Thiamine Biosynthesis Superpathways | |
| PWY-7664 | oleate biosynthesis IV (anaerobic) | Biosynthesis → Fatty Acid and Lipid Biosynthesis → Fatty Acid Biosynthesis → Unsaturated Fatty Acid Biosynthesis | | |
| PWY-6969 | TCA cycle V (2-oxoglutarate synthase) | Generation of Precursor Metabolites and Energy → TCA cycle | | |
| PWY-6507 | 4-deoxy-L-threo-hex-4-enopyranuronate degradation | Degradation/Utilization/Assimilation → Secondary Metabolite Degradation → Sugar Derivative Degradation | | |
| PWY-6282 | palmitoleate biosynthesis I (from (5Z)-dodec-5-enoate) | Biosynthesis → Fatty Acid and Lipid Biosynthesis → Fatty Acid Biosynthesis → Unsaturated Fatty Acid Biosynthesis | | |
| PWY-5154 | L-arginine biosynthesis III (via N-acetyl-L-citrulline) | Biosynthesis → Amino Acid Biosynthesis → Proteinogenic Amino Acid Biosynthesis | | |
| PWYG-321 | mycolate biosynthesis | Biosynthesis → Fatty Acid and Lipid Biosynthesis → Fatty Acid Biosynthesis | | |
| PWY-6612 | superpathway of tetrahydrofolate biosynthesis | Biosynthesis → Cofactor, Carrier, and Vitamin Biosynthesis → Carrier Biosynthesis → Single Carbon Carrier Biosynthesis → Folate Biosynthesis | Biosynthesis → Cofactor, Carrier, and Vitamin Biosynthesis → Vitamin Biosynthesis → Folate Biosynthesis Superpathways | |
| PWY-5989 | stearate biosynthesis II (bacteria and plants) | Biosynthesis → Fatty Acid and Lipid Biosynthesis → Fatty Acid Biosynthesis → Stearate Biosynthesis | | |
| PWY-6519 | 8-amino-7-oxononanoate biosynthesis I | Biosynthesis → Other Biosynthesis → 8-Amino-7-oxononanoate Biosynthesis | | |

| | | | | |
|---|---|---|---|---|
| BIOTIN-BIOSYNTHESIS-PWY | biotin biosynthesis I | Biosynthesis → Cofactor, Carrier, and Vitamin Biosynthesis → Enzyme Cofactor Biosynthesis → Biotin Biosynthesis | Superpathways | |
| P108-PWY | pyruvate fermentation to propanoate I | Generation of Precursor Metabolites and Energy → Fermentation → Fermentation of Pyruvate → Pyruvate Fermentation to Propanoate | Generation of Precursor Metabolites and Energy → Fermentation → Fermentation to Short-Chain Fatty Acids → Fermentation to Propanoate → Pyruvate Fermentation to Propanoate | |
| PRPP-PWY | superpathway of histidine, purine, and pyrimidine biosynthesis | Superpathways | | |
| PWY-7242 | D-fructuronate degradation | Degradation/Utilization/Assimilation → Carboxylate Degradation → Sugar Acid Degradation | Degradation/Utilization/Assimilation → Secondary Metabolite Degradation → Sugar Derivative Degradation → Sugar Acid Degradation | |
| FASYN-INITIAL-PWY | superpathway of fatty acid biosynthesis initiation (E. coli) | Biosynthesis → Fatty Acid and Lipid Biosynthesis → Fatty Acid Biosynthesis | Superpathways | |
| FOLSYN-PWY | superpathway of tetrahydrofolate biosynthesis and salvage | Biosynthesis → Cofactor, Carrier, and Vitamin Biosynthesis → Carrier Biosynthesis → Single Carbon Carrier Biosynthesis → Folate Biosynthesis | Biosynthesis → Cofactor, Carrier, and Vitamin Biosynthesis → Vitamin Biosynthesis → Folate Biosynthesis | Superpathways |
| PWY0-845 | superpathway of pyridoxal 5'-phosphate biosynthesis and salvage | Biosynthesis → Cofactor, Carrier, and Vitamin Biosynthesis → Enzyme Cofactor Biosynthesis → Vitamin B6 BiosynthesisBiosynthesis → Cofactor, Carrier, and Vitamin Biosynthesis → Vitamin Biosynthesis → Vitamin B6 BiosynthesisSuperpathways | Biosynthesis → Cofactor, Carrier, and Vitamin Biosynthesis → Vitamin Biosynthesis → Vitamin B6 Biosynthesis | Superpathways |
| GLUCONEO-PWY | gluconeogenesis I | Biosynthesis → Carbohydrate Biosynthesis → Sugar Biosynthesis → Gluconeogenesis | | |
| PWY-1269 | CMP-3-deoxy-D-manno-octulosonate biosynthesis | Biosynthesis → Carbohydrate Biosynthesis → Sugar Biosynthesis → Sugar Nucleotide Biosynthesis → CMP-sugar Biosynthesis → CMP-3-deoxy-D-manno-octulosonate Biosynthesis | | |

| | | | | |
|---|---|---|---|---|
| PWY-6703 | preQ0 biosynthesis | Biosynthesis → Secondary Metabolite Biosynthesis | | |
| PWY-5695 | inosine 5'-phosphate degradation | Degradation/Utilization/Assimilation → Nucleoside and Nucleotide Degradation → Purine Nucleotide Degradation | | |
| NAGLIPASYN-PWY | lipid IVA biosynthesis (E. coli) | Biosynthesis → Cell Structure Biosynthesis → Lipopolysaccharide Biosynthesis | Biosynthesis → Fatty Acid and Lipid Biosynthesis | |
| PWY-6467 | Kdo transfer to lipid IVA III (Chlamydia) | Biosynthesis → Cell Structure Biosynthesis → Lipopolysaccharide Biosynthesis → Kdo Transfer to Lipid IVA | Superpathways | |
| COLANSYN-PWY | colanic acid building blocks biosynthesis | Biosynthesis → Carbohydrate Biosynthesis | Superpathways | |
| PYRIDOXSYN-PWY | pyridoxal 5'-phosphate biosynthesis I | Biosynthesis → Cofactor, Carrier, and Vitamin Biosynthesis → Enzyme Cofactor Biosynthesis → Vitamin B6 Biosynthesis | Biosynthesis → Cofactor, Carrier, and Vitamin Biosynthesis → Vitamin Biosynthesis → Vitamin B6 Biosynthesis | |
| RHAMCAT-PWY | L-rhamnose degradation I | Degradation/Utilization/Assimilation → Carbohydrate Degradation → Sugar Degradation → L-rhamnose Degradation | | |
| GALACTUROCAT-PWY | D-galacturonate degradation I | Degradation/Utilization/Assimilation → Carboxylate Degradation → Sugar Acid Degradation → D-Galacturonate Degradation | Degradation/Utilization/Assimilation → Secondary Metabolite Degradation → Sugar Derivative Degradation → Sugar Acid Degradation → D-Galacturonate Degradation | |
| PWY-6147 | 6-hydroxymethyl-dihydropterin diphosphate biosynthesis I | Biosynthesis → Cofactor, Carrier, and Vitamin Biosynthesis → Carrier Biosynthesis → Single Carbon Carrier Biosynthesis → Folate Biosynthesis → 6-Hydroxymethyl-Dihydropterin Diphosphate | Biosynthesis → Cofactor, Carrier, and Vitamin Biosynthesis → Vitamin Biosynthesis → Folate Biosynthesis → 6-Hydroxymethyl-Dihydropterin Diphosphate Biosynthesis | |

| | | | | |
|---|---|---|---|---|
| PWY-7539 | 6-hydroxymethyl-dihydropterin diphosphate biosynthesis III (Chlamydia) | Biosynthesis → Cofactor, Carrier, and Vitamin Biosynthesis → Carrier Biosynthesis → Single Carbon Carrier Biosynthesis → Folate Biosynthesis → 6-Hydroxymethyl-Dihydropterin Diphosphate | Biosynthesis → Cofactor, Carrier, and Vitamin Biosynthesis → Vitamin Biosynthesis → Folate Biosynthesis → 6-Hydroxymethyl-Dihydropterin Diphosphate Biosynthesis | |
| PWY-7254 | TCA cycle VII (acetate-producers) | Generation of Precursor Metabolites and Energy → TCA cycle | | |
| PWY-5918 | superpathway of heme b biosynthesis from glutamate | Biosynthesis → Cofactor, Carrier, and Vitamin Biosynthesis → Enzyme Cofactor Biosynthesis → Heme Biosynthesis → Heme b Biosynthesis | Biosynthesis → Tetrapyrrole Biosynthesis → Porphyrin Compound Biosynthesis → Heme Biosynthesis → Heme b Biosynthesis | Superpathways |
| PWY0-1241 | ADP-L-glycero-β-D-manno-heptose biosynthesis | Biosynthesis → Carbohydrate Biosynthesis → Sugar Biosynthesis → Sugar Nucleotide Biosynthesis → ADP-sugar Biosynthesis | | |
| PWY-7200 | superpathway of pyrimidine deoxyribonucleoside salvage | Biosynthesis → Nucleoside and Nucleotide Biosynthesis → Pyrimidine Nucleotide Biosynthesis → Pyrimidine Nucleotide Salvage | Superpathways | |
| PWY-5005 | biotin biosynthesis II | Biosynthesis → Cofactor, Carrier, and Vitamin Biosynthesis → Enzyme Cofactor Biosynthesis → Biotin Biosynthesis | | |
| HISDEG-PWY | L-histidine degradation I | Degradation/Utilization/Assimilation → Amino Acid Degradation → Proteinogenic Amino Acid Degradation → L-histidine Degradation | | |
| PWY-6478 | GDP-D-glycero-α-D-manno-heptose biosynthesis | Biosynthesis → Carbohydrate Biosynthesis → Sugar Biosynthesis → Sugar Nucleotide Biosynthesis → GDP-sugar Biosynthesis | | |
| PWY0-1415 | superpathway of heme b biosynthesis from uroporphyrinogen-III | Biosynthesis → Cofactor, Carrier, and Vitamin Biosynthesis → Enzyme Cofactor Biosynthesis → Heme Biosynthesis → Heme b Biosynthesis | Biosynthesis → Tetrapyrrole Biosynthesis → Porphyrin Compound Biosynthesis → Heme Biosynthesis → Heme b Biosynthesis | Superpathways |
| PWY-7255 | ergothioneine biosynthesis I (bacteria) | Biosynthesis → Secondary Metabolite Biosynthesis → Ergothioneine Biosynthesis | | |

| | | | | |
|---|---|---|---|---|
| PWY-6163 | chorismate biosynthesis from 3-dehydroquinate | Biosynthesis → Aromatic Compound Biosynthesis → Chorismate Biosynthesis | | |
| DAPLYSINESYN-PWY | L-lysine biosynthesis I | Biosynthesis → Amino Acid Biosynthesis → Proteinogenic Amino Acid Biosynthesis → L-lysine Biosynthesis | | |
| P164-PWY | purine nucleobases degradation I (anaerobic) | Degradation/Utilization/Assimilation → Nucleoside and Nucleotide Degradation → Purine Nucleotide Degradation | Generation of Precursor Metabolites and Energy → Fermentation | |
| PWY-7221 | guanosine ribonucleotides de novo biosynthesis | Biosynthesis → Nucleoside and Nucleotide Biosynthesis → Purine Nucleotide Biosynthesis → Purine Nucleotide De Novo Biosynthesis → Purine Riboucleotide De Novo Biosynthesis | | |
| PWY-5686 | UMP biosynthesis I | Biosynthesis → Nucleoside and Nucleotide Biosynthesis → Pyrimidine Nucleotide Biosynthesis → Pyrimidine Nucleotide De Novo Biosynthesis → Pyrimidine Ribonucleotide De Novo Biosynthesis → UMP Biosynthesis | | |
| COA-PWY | coenzyme A biosynthesis I (prokaryotic) | Biosynthesis → Cofactor, Carrier, and Vitamin Biosynthesis → Carrier Biosynthesis → Coenzyme A Biosynthesis | | |
| ARO-PWY | chorismate biosynthesis I | Biosynthesis → Aromatic Compound Biosynthesis → Chorismate Biosynthesis | Superpathways | |
| PWY-6749 | CMP-legionaminate biosynthesis I | Biosynthesis → Carbohydrate Biosynthesis → Sugar Biosynthesis → Sugar Nucleotide Biosynthesis → CMP-sugar Biosynthesis → CMP-legionaminate biosynthesi | | |
| ANAGLYCOLYSIS-PWY | glycolysis III (from glucose) | Generation of Precursor Metabolites and Energy → Glycolysis | | |
| PWY-6121 | 5-aminoimidazole ribonucleotide biosynthesis I | Biosynthesis → Nucleoside and Nucleotide Biosynthesis → Purine Nucleotide Biosynthesis → 5-Aminoimidazole Ribonucleotide Biosynthesis | | |
| COMPLETE-ARO-PWY | superpathway of aromatic amino acid biosynthesis | Biosynthesis → Amino Acid Biosynthesis | Superpathways | |
| THRESYN-PWY | superpathway of L-threonine biosynthesis | Biosynthesis → Amino Acid Biosynthesis → Proteinogenic Amino Acid Biosynthesis → L-threonine Biosynthesis | Superpathways | |
| PWY0-1319 | CDP-diacylglycerol biosynthesis II | Biosynthesis → Fatty Acid and Lipid Biosynthesis → Phospholipid Biosynthesis → CDP-diacylglycerol Biosynthesis | | |
| PWY-5667 | CDP-diacylglycerol biosynthesis I | Biosynthesis → Fatty Acid and Lipid Biosynthesis → Phospholipid Biosynthesis → CDP-diacylglycerol Biosynthesis | | |

| | | | | |
|---|---|---|---|---|
| CALVIN-PWY | Calvin-Benson-Bassham cycle | Biosynthesis → Carbohydrate Biosynthesis → Sugar Biosynthesis | Degradation/Utilization/Assimilation → C1 Compound Utilization and Assimilation → CO2 Fixation → Autotrophic CO2 Fixation | |
| PWY-6385 | peptidoglycan biosynthesis III (mycobacteria) | Biosynthesis → Cell Structure Biosynthesis → Cell Wall Biosynthesis → Peptidoglycan BiosynthesisSuperpathways | Superpathways | |
| PWY-6277 | superpathway of 5-aminoimidazole ribonucleotide biosynthesis | Biosynthesis → Nucleoside and Nucleotide Biosynthesis → Purine Nucleotide Biosynthesis → 5-Aminoimidazole Ribonucleotide Biosynthesis | Superpathways | |
| PWY-6122 | 5-aminoimidazole ribonucleotide biosynthesis II | Biosynthesis → Nucleoside and Nucleotide Biosynthesis → Purine Nucleotide Biosynthesis → 5-Aminoimidazole Ribonucleotide Biosynthesis | | |
| PWY-7219 | adenosine ribonucleotides de novo biosynthesis | Biosynthesis → Nucleoside and Nucleotide Biosynthesis → Purine Nucleotide | | |
| PWY-6386 | UDP-N-acetylmuramoyl-pentapeptide biosynthesis II (lysine-containing) | Biosynthesis → Cell Structure Biosynthesis → Cell Wall Biosynthesis → UDP-N-Acetylmuramoyl-Pentapeptide Biosynthesis | | |
| COBALSYN-PWY | superpathway of adenosylcobalamin salvage from cobinamide I | Biosynthesis → Cofactor, Carrier, and Vitamin Biosynthesis → Enzyme Cofactor Biosynthesis → Cobamide Biosynthesis → Cobinamide Salvage → Adenosylcobalamin Salvage from Cobinamide | Superpathways | |
| PWY-6126 | superpathway of adenosine nucleotides de novo biosynthesis II | Biosynthesis → Nucleoside and Nucleotide Biosynthesis → Purine Nucleotide Biosynthesis → Purine Nucleotide De Novo Biosynthesis | Superpathways | |
| PWY-5384 | sucrose degradation IV (sucrose phosphorylase) | Degradation/Utilization/Assimilation → Carbohydrate Degradation → Sugar Degradation → Sucrose Degradation | | |
| PWY-7229 | superpathway of adenosine nucleotides de novo biosynthesis I | Biosynthesis → Nucleoside and Nucleotide Biosynthesis → Purine Nucleotide Biosynthesis → Purine Nucleotide De Novo Biosynthesis | Superpathways | |
| PWY0-1298 | superpathway of pyrimidine deoxyribonucleosides degradation | Degradation/Utilization/Assimilation → Nucleoside and Nucleotide Degradation → Pyrimidine Nucleotide Degradation | Superpathways | |

| | | | | |
|---|---|---|---|---|
| PWY-7208 | superpathway of pyrimidine nucleobases salvage | Biosynthesis → Nucleoside and Nucleotide Biosynthesis → Pyrimidine Nucleotide Biosynthesis → Pyrimidine Nucleotide SalvageSuperpathways | Superpathways | |
| PWY-5100 | pyruvate fermentation to acetate and lactate II | Generation of Precursor Metabolites and Energy → Fermentation → Fermentation of Pyruvate → pyruvate fermentation to lactate | Generation of Precursor Metabolites and Energy → Fermentation → Fermentation of Pyruvate → Pyruvate Fermentation to Acetate | Degradation/Utilization/Assimilation → Carboxylate Degradation → Fermentation to Acetate → Pyruvate Fermentation to Acetate |
| REDCITCYC | TCA cycle VI (Helicobacter) | Generation of Precursor Metabolites and Energy → TCA cycle | | |
| TRNA-CHARGING-PWY | tRNA charging | Biosynthesis → Aminoacyl-tRNA Charging | Metabolic Clusters | |
| PWY-3001 | superpathway of L-isoleucine biosynthesis I | Biosynthesis → Amino Acid Biosynthesis → Proteinogenic Amino Acid Biosynthesis → L-isoleucine Biosynthesis | Superpathways | |
| OANTIGEN-PWY | O-antigen building blocks biosynthesis (E. coli) | Biosynthesis → Carbohydrate Biosynthesis → Sugar Biosynthesis → Sugar Nucleotide Biosynthesi | Metabolic Clusters | Superpathways |
| PWY-5103 | L-isoleucine biosynthesis III | Biosynthesis → Amino Acid Biosynthesis → Proteinogenic Amino Acid Biosynthesis → L-isoleucine Biosynthesis | | |
| PWY-5676 | acetyl-CoA fermentation to butanoate II | Generation of Precursor Metabolites and Energy → Fermentation → Fermentation to Short-Chain Fatty Acids → Fermentation to Butanoate | Superpathways | |
| PHOSLIPSYN-PWY | superpathway of phospholipid biosynthesis I (bacteria) | Biosynthesis → Fatty Acid and Lipid Biosynthesis → Phospholipid Biosynthesis | Superpathways | |
| PEPTIDOGLYCANSYN-PWY | peptidoglycan biosynthesis I (meso-diaminopimelate containing) | Biosynthesis → Cell Structure Biosynthesis → Cell Wall Biosynthesis → Peptidoglycan Biosynthesis | Superpathways | |
| PWY-6387 | UDP-N-acetylmuramoyl-pentapeptide biosynthesis I (meso-diaminopimelate containing) | Biosynthesis → Cell Structure Biosynthesis → Cell Wall Biosynthesis → UDP-N-Acetylmuramoyl-Pentapeptide Biosynthesis | | |
| PWY4FS-8 | phosphatidylglycerol biosynthesis II (non-plastidic) | Biosynthesis → Fatty Acid and Lipid Biosynthesis → Phospholipid Biosynthesis → Phosphatidylglycerol Biosynthesis | Superpathways | |

| | | | | |
|---|---|---|---|---|
| PWY4FS-7 | phosphatidylglycerol biosynthesis I (plastidic) | Biosynthesis → Fatty Acid and Lipid<br>Biosynthesis → Phospholipid<br>Biosynthesis → Phosphatidylglycerol Biosynthesis | Superpathways | |
| PWY-6471 | peptidoglycan biosynthesis IV (Enterococcus faecium) | Biosynthesis → Cell Structure Biosynthesis → Cell Wall Biosynthesis → Peptidoglycan Biosynthesis<br>Detoxification → Antibiotic Resistance | Superpathways | |
| VALSYN-PWY | L-valine biosynthesis | Biosynthesis → Amino Acid<br>Biosynthesis → Proteinogenic Amino Acid<br>Biosynthesis → L-valine Biosynthesis | | |
| ILEUSYN-PWY | L-isoleucine biosynthesis I (from threonine) | Biosynthesis → Amino Acid<br>Biosynthesis → Proteinogenic Amino Acid<br>Biosynthesis → L-isoleucine Biosynthesis | | |
| HISTSYN-PWY | L-histidine biosynthesis | Biosynthesis → Amino Acid<br>Biosynthesis → Proteinogenic Amino Acid<br>Biosynthesis → L-histidine Biosynthesis | | |
| NONMEVIPP-PWY | methylerythritol phosphate pathway I | Biosynthesis → Secondary Metabolite<br>Biosynthesis → Terpenoid | Biosynthesis → Hemiterpene<br>Biosynthesis → Isopentenyl Diphosphate | Biosynthesis → Methylerythritol Phosphate Pathways |
| PWY-7560 | methylerythritol phosphate pathway II | Biosynthesis → Secondary Metabolite<br>Biosynthesis → Terpenoid | Biosynthesis → Hemiterpene<br>Biosynthesis → Isopentenyl Diphosphate | Biosynthesis → Methylerythritol Phosphate Pathways |
| PWY-5121 | superpathway of geranylgeranyl diphosphate biosynthesis II (via MEP) | Biosynthesis → Polyprenyl<br>Biosynthesis → Geranylgeranyl Diphosphate Biosynthesis | Biosynthesis → Secondary Metabolite<br>Biosynthesis → Terpenoid | Biosynthesis → Diterpenoid Biosynthesis |
| BRANCHED-CHAIN-AA-SYN-PWY | superpathway of branched chain amino acid biosynthesis | Biosynthesis → Amino Acid Biosynthesis | Superpathways | |
| PWY-5104 | L-isoleucine biosynthesis IV | Biosynthesis → Amino Acid<br>Biosynthesis → Proteinogenic Amino Acid<br>Biosynthesis → L-isoleucine Biosynthesis | | |
| PWY-5101 | L-isoleucine biosynthesis II | Biosynthesis → Amino Acid<br>Biosynthesis → Proteinogenic Amino Acid<br>Biosynthesis → L-isoleucine Biosynthesis | | |
| PWY-7111 | pyruvate fermentation to isobutanol (engineered) | Generation of Precursor Metabolites and Energy → Fermentation → Fermentation of Pyruvate | Generation of Precursor Metabolites and Energy → Fermentation → Fermentation to Alcohols | |
| UDPNAGSYN-PWY | UDP-N-acetyl-D-glucosamine biosynthesis I | Biosynthesis → Carbohydrate Biosynthesis → Sugar Biosynthesis → Sugar Nucleotide<br>Biosynthesis → UDP-sugar Biosynthesis → UDP-N-acetyl-D-glucosamine Biosynthesis | | |

| | | | | |
|---|---|---|---|---|
| P161-PWY | acetylene degradation (anaerobic) | Degradation/Utilization/Assimilation → Carboxylate Degradation → Fermentation to Acetate | Generation of Precursor Metabolites and Energy → Fermentation → Fermentation of Pyruvate → Pyruvate Fermentation to Ethanol | Generation of Precursor Metabolites and Energy → Fermentation → Fermentation to Alcohols → Pyruvate Fermentation to Ethanol |
| PWY0-1586 | peptidoglycan maturation (meso-diaminopimelate containing) | Biosynthesis → Cell Structure Biosynthesis → Cell Wall Biosynthesis → Peptidoglycan Biosynthesis | | |
| GLYCOLYSIS-E-D | superpathway of glycolysis and the Entner-Doudoroff pathway | Generation of Precursor Metabolites and Energy | Superpathways | |
| SALVADEHYPOX-PWY | adenosine nucleotides degradation II | Degradation/Utilization/Assimilation → Nucleoside and Nucleotide Degradation → Purine Nucleotide Degradation → Adenosine Nucleotide Degradation | | |
| PWY-3781 | aerobic respiration I (cytochrome c) | Generation of Precursor Metabolites and Energy → Electron Transfer Chains | Generation of Precursor Metabolites and Energy → Respiration → Aerobic Respiration | |
| P441-PWY | superpathway of N-acetylneuraminate degradation | Degradation/Utilization/Assimilation → Carboxylate Degradation | Superpathways | |
| P23-PWY | reductive TCA cycle I | Degradation/Utilization/Assimilation → C1 Compound Utilization and Assimilation → CO2 Fixation → Autotrophic CO2 Fixation → Reductive TCA Cycles | | |
| PWY-6608 | guanosine nucleotides degradation III | Degradation/Utilization/Assimilation → Nucleoside and Nucleotide Degradation → Purine Nucleotide Degradation | Superpathways | |
| PWY-6353 | purine nucleotides degradation II (aerobic) | Degradation/Utilization/Assimilation → Nucleoside and Nucleotide Degradation → Purine Nucleotide Degradation | Superpathways | |
| METHYLGALLATE-DEGRADATION-PWY | methylgallate degradation | Degradation/Utilization/Assimilation → Aromatic Compound Degradation | | |
| GALLATE-DEGRADATION-I-PWY | gallate degradation II | Degradation/Utilization/Assimilation → Aromatic Compound Degradation → Gallate Degradation | | |
| PWY-7663 | gondoate biosynthesis (anaerobic) | Biosynthesis → Fatty Acid and Lipid Biosynthesis → Fatty Acid Biosynthesis → Unsaturated Fatty Acid Biosynthesis | | |

| | | | | |
|---|---|---|---|---|
| PWY-6629 | superpathway of L-tryptophan biosynthesis | Biosynthesis → Amino Acid<br>Biosynthesis → Proteinogenic Amino Acid<br>Biosynthesis → L-tryptophan Biosynthesis | Superpathways | |
| GALLARATE-DEGRADATION-II-PWY | gallate degradation I | Degradation/Utilization/Assimilation → Aromatic Compound Degradation → Gallate Degradation | | |
| PWY-6590 | superpathway of Clostridium acetobutylicum acidogenic fermentation | Generation of Precursor Metabolites and Energy → Fermentation → Fermentation of Pyruvate | Superpathways | |
| P562-PWY | myo-inositol degradation I | Degradation/Utilization/Assimilation → Secondary Metabolite Degradation → Sugar Derivative Degradation → Sugar Alcohol Degradation | | |
| PWY-5913 | partial TCA cycle (obligate autotrophs) | Generation of Precursor Metabolites and Energy → TCA cycle | | |
| PWY0-781 | aspartate superpathway | Superpathways | | |
| P122-PWY | heterolactic fermentation | Fermentation to Lactic Acid | Generation of Precursor Metabolites and Energy → Fermentation → Fermentation to Alcohols → Pyruvate Fermentation to Ethanol | Generation of Precursor Metabolites and Energy → Fermentation → Fermentation of Pyruvate → Pyruvate Fermentation to Ethanol |
| PWY-7234 | inosine-5'-phosphate biosynthesis III | Biosynthesis → Nucleoside and Nucleotide<br>Biosynthesis → Purine Nucleotide<br>Biosynthesis → Purine Nucleotide De Novo<br>Biosynthesis → Purine Riboucleotide De Novo<br>Biosynthesis → Inosine-5'-phosphate Biosynthesis | | |
| PWY-6470 | peptidoglycan biosynthesis V (β-lactam resistance) | Biosynthesis → Cell Structure Biosynthesis → Cell Wall Biosynthesis → Peptidoglycan Biosynthesis | | |
| PWY-6317 | D-galactose degradation I (Leloir pathway) | Degradation/Utilization/Assimilation → Carbohydrate Degradation → Sugar Degradation → Galactose Degradation | | |
| PWY-7237 | myo-, chiro- and scyllo-inositol degradation | Degradation/Utilization/Assimilation → Secondary Metabolite Degradation → Sugar Derivative Degradation → Sugar Alcohol Degradation | Superpathways | |
| GLCMANNANAUT-PWY | superpathway of N-acetylglucosamine, N-acetylmannosamine and N-acetylneuraminate degradation | Degradation/Utilization/Assimilation → Amine and Polyamine Degradation | Superpathways | |

| | | | | |
|---|---|---|---|---|
| PWY-6151 | S-adenosyl-L-methionine salvage I | Biosynthesis → Cofactor, Carrier, and Vitamin Biosynthesis → Enzyme Cofactor Biosynthesis → S-adenosyl-L-methionine Biosynthesis | | |
| PWY-6892 | thiazole component of thiamine diphosphate biosynthesis I | Biosynthesis → Cofactor, Carrier, and Vitamin Biosynthesis → Enzyme Cofactor Biosynthesis → Thiamine Biosynthesis → Thiazole Biosynthesis | Biosynthesis → Cofactor, Carrier, and Vitamin Biosynthesis → Vitamin Biosynthesis → Thiamine Biosynthesis → Thiazole Biosynthesis | |
| PYRIDNUCSAL-PWY | NAD salvage pathway I (PNC VI cycle) | Biosynthesis → Cofactor, Carrier, and Vitamin Biosynthesis → Carrier Biosynthesis → Electron Carrier Biosynthesis → NAD Metabolism → NAD Biosynthesis | Biosynthesis → Cofactor, Carrier, and Vitamin Biosynthesis → Enzyme Cofactor Biosynthesis → NAD Biosynthesis | Biosynthesis → Cofactor, Carrier, and Vitamin Biosynthesis → Enzyme Cofactor Biosynthesis → NAD Metabolism → NAD Biosynthesis |
| FERMENTATION-PWY | mixed acid fermentation | Generation of Precursor Metabolites and Energy → Fermentation → Fermentation of Pyruvate → Pyruvate Fermentation to Ethanol | Generation of Precursor Metabolites and Energy → Fermentation → Fermentation to Alcohols → Pyruvate Fermentation to Ethanol | Generation of Precursor Metabolites and Energy → Fermentation → Fermentation to Short-Chain Fatty Acids |
| PWY-6737 | starch degradation V | Degradation/Utilization/Assimilation → Carbohydrate Degradation → Polysaccharide Degradation → Starch Degradation | | |
| GLYCOLYSIS | glycolysis I (from glucose 6-phosphate) | Generation of Precursor Metabolites and Energy → Glycolysis | | |
| PWY-5484 | glycolysis II (from fructose 6-phosphate) | Generation of Precursor Metabolites and Energy → Glycolysis | | |
| GLYCOCAT-PWY | glycogen degradation I | Degradation/Utilization/Assimilation → Carbohydrate Degradation → Polysaccharide Degradation → Glycan Degradation | | |
| ANAEROFRUCAT-PWY | homolactic fermentation | Generation of Precursor Metabolites and Energy → Fermentation → Fermentation to Short-Chain Fatty Acids → Fermentation to Lactate | Superpathways | |

| | | | | |
|---|---|---|---|---|
| P124-PWY | Bifidobacterium shunt | Generation of Precursor Metabolites and Energy → Fermentation → Fermentation to Short-Chain Fatty Acids → Fermentation to Lactate | Generation of Precursor Metabolites and Energy → Fermentation → Fermentation to Short-Chain Fatty Acids → Fermentation to Acetate | Degradation/Utilization/Assimilation → Carboxylate Degradation → Fermentation to Acetate |
| P461-PWY | hexitol fermentation to lactate, formate, ethanol and acetate | Generation of Precursor Metabolites and Energy → Fermentation → Fermentation to Short-Chain Fatty Acids → Fermentation to Lactate | Degradation/Utilization/Assimilation → Carboxylate Degradation → Fermentation to Acetate | |
| PWY-621 | sucrose degradation III (sucrose invertase) | Degradation/Utilization/Assimilation → Carbohydrate Degradation → Sugar Degradation → Sucrose Degradation | | |
| PWY0-1061 | superpathway of L-alanine biosynthesis | Biosynthesis → Amino Acid Biosynthesis → Proteinogenic Amino Acid Biosynthesis → L-alanine Biosynthesis | Superpathways | |

**Table S1. MetaCyc pathway and superclass information for the discriminating pathways between platinum-resistant and platinum-sensitive EOC patients receiving neoadjuvant or adjuvant chemotherapy.** Differentially represented inferred functions were identified by linear discriminant analysis (LDA) effect size (LEfSe) analysis. See also **Figure 7**.
